# Cost‐Efficiency and Cost‐Effectiveness of Alternative Bouillon Fortification Programs: Evidence for Burkina Faso, Nigeria, and Senegal

**DOI:** 10.1111/nyas.70137

**Published:** 2025-12-09

**Authors:** Stephen A. Vosti, Michael Jarvis, Lauren Thompson, Emily Becher, Maguette Beye, Faith Ishaya, Karim Koudougou, Reina Engle‐Stone, Katherine P. Adams

**Affiliations:** ^1^ Institute for Global Nutrition University of California, Davis Davis California USA; ^2^ Department of Agricultural and Resource Economics University of California, Davis Davis California USA; ^3^ Department of Nutrition University of California, Davis Davis California USA; ^4^ Helen Keller International Dakar Senegal; ^5^ Helen Keller International Abuja Nigeria; ^6^ Helen Keller International Ouagadougou Burkina Faso

**Keywords:** bouillon, Burkina Faso, cost‐effectiveness, costs, large‐scale food fortification, Nigeria, Senegal

## Abstract

Cost‐efficiency and cost‐effectiveness of fortification programs are not single, fixed numbers, but rather depend on dietary intake patterns and choices regarding the numbers and amounts of fortificants in premixes. We modeled the cost‐efficiency and cost‐effectiveness of alternative bouillon fortification formulations with vitamins A, B9, and B12, iron, and zinc in Burkina Faso, Nigeria, and Senegal. Cost per consumer reached varied by nutrient and context; for example, cost‐efficiency for vitamin A (96 µg/g) ranged from ∼$0.05 (Burkina Faso) to ∼$0.12 (Nigeria). In Nigeria, the cost per woman effectively covered by vitamin A‐fortified bouillon delivering 40−250 µg/g ranged from ∼$2.00 to ∼$6.50; in Burkina Faso, this metric fell from ∼$3.25 to ∼$1.25 for the same programs. Cost per child‐life saved by bouillon co‐fortified with vitamin A (96 µg/g), zinc (1.68 mg/g), and folic acid (28.8 µg/g) ranged from ∼$1272 (Burkina Faso) to ∼$3234 (Senegal). Cost functions tended to be linear in the amounts of fortificants in premixes; nutritional and child mortality benefit curves tended to be nonlinear, with context‐specific shapes. Identifying fortification levels at which marginal benefits become small could help inform the design of efficient programs; design should also consider technical issues, program objectives, affordability, and sustainability.

## Graphical abstract

Achieving dietary adequacy in vitamin A, zinc, and/or folate can save child‐lives. Different amounts and combinations of micronutrients have different costs and different effects on child mortality. This study presents ten bouillon fortification options for Nigeria. Scenarios 7 and 8 are the most cost‐effective, but save relatively few child‐lives. Scenario 9 is the least cost‐effective. Scenarios 1, 3, 6, and 10 are similarly cost‐effective; Scenario 1 saves the most child‐lives. This information is relevant for policy discussions.

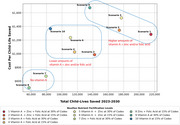

## 1 Introduction

Micronutrient deficiencies remain a major public health concern in most low‐ and middle‐income countries (LMICs) [1, 2], with important consequences for child health and development [3], including, for some micronutrients, child mortality [4]. Dietary inadequacies are major contributors to these deficiencies [3] and are also prevalent in LMICs [5], but these inadequacies also provide entry points for addressing micronutrient deficiencies by increasing the micronutrient content of diets at a large scale via food and condiment fortification, biofortification, and agronomic biofortification programs, and at a smaller and more targeted scale via broad‐based and in‐home fortification and supplementation programs [6]. Indeed, some large‐scale food fortification (LSFF) programs have been shown to be impactful and cost‐effective [7−9]. However, in some cases, even combinations of currently available LSFF programs cannot completely address micronutrient inadequacies, either because chosen micronutrient delivery vehicles (alternative foods and/or condiments) are not consumed at all or in sufficient quantities by those in need, or the amounts of micronutrients contained in these vehicles are small relative to dietary requirements [10−13]. The latter can be a consequence of fortification standards that do not “match” the nutritional needs of target populations, the failure of industry to fortify to standards, or some combination of the two [9]. Therefore, there is a need in many LMIC settings for new, impactful, and equitable delivery vehicles for micronutrients of public health concern.

There is a recent renewed interest in the potential for fortified bouillon products^1^ to reduce dietary micronutrient inadequacies [14, 15]. In the context of many West African countries, bouillon is an excellent candidate micronutrient delivery vehicle for several reasons. First, the geographic and socioeconomic reach of bouillon is second only to salt, for example, nearly the entire population of Nigeria, including the rural poor, consumes bouillon on a daily basis in soups, stews, and so on [16]. Second, although some technical challenges exist, the bouillon matrix itself can accommodate multiple micronutrients in substantial amounts [17]. Third, modeled evidence from several West African countries suggests that the nutritional benefits of fortified bouillon can be substantial for children and for women of reproductive age (WRA) [11−13]; the same is true for selected micronutrients on child mortality [4]. Modeled evidence on bouillon fortification program costs suggests that start‐up costs for government and for industry can be substantial, but that the main driver of bouillon fortification program costs will be premix costs, which will depend largely on the types and amounts of fortificants included in premixes, and on the size and bouillon consumption habits of national populations [18]. Modeled evidence also suggests that fortified bouillon can be a cost‐effective component of an overall LSFF program strategy in some contexts [19, 20]. All that said, technical and other challenges remain in developing and marketing commercially viable multifortified bouillon, and in determining how best to integrate bouillon fortification programs into national sodium reduction strategies and programs [21].

As interest rises in the potential for harnessing the reach of bouillon for addressing micronutrient inadequacies, questions arise regarding the details of alternative fortification programs. More specifically, if bouillon is chosen as a new vehicle for conveying micronutrients, which micronutrients should be included in it and at what levels? While there are guidance documents and many examples of more traditional food and condiment vehicles, for example, wheat flour, sugar, edible oils, salt, and so on, that policymakers in LMICs can look to for guidance on developing similar programs [22], the same is not true for bouillon. Indeed, while there are examples of voluntary bouillon fortification by industry with single micronutrients [23], to date, there is only one national voluntary standard for bouillon fortification, with Nigeria being the pathbreaker in this case [24, 25].

Alongside rising interest in fortifying bouillon is the important, broader issue of sodium consumption in LMICs [26]. While bouillon cubes commonly contain roughly 50% sodium by weight [14], recent studies have shown that bouillon's overall contribution to sodium intake is modest (e.g., ∼15% in Senegal [27]) and there is no evidence to suggest that fortification per se would increase bouillon consumption. All that said, bouillon (fortified or not) should be included in broader national policy discussions around reducing sodium intake and the policy actions required to do so [28].

In the absence of guidance on the design of bouillon fortification programs, policymakers and those who advise them are called upon to reflect on an array of criteria that will influence their decisions. Chief among these criteria are technical and commercial viability [21], affordability by all stakeholders [29], sustainability [9], and efficiency as stand‐alone interventions and vis‐à‐vis other programmatic options for addressing micronutrient inadequacies [19, 29].

This paper uses modeled evidence from Senegal, Nigeria, and Burkina Faso to examine the last of these criteria, efficiency, which is essential for resource‐constrained policymakers to consider in any setting, but especially in the contexts of LMICs. We focus narrowly on bouillon as a micronutrient delivery vehicle, but we provide broad sets of estimates of the potential impacts and costs of bouillon fortification programs under alternative assumptions regarding the amounts of five micronutrients (vitamin A, vitamin B12, folic acid, iron, and zinc) that could be included in premixes. Different assumptions yield different estimates of levels of cost‐effectiveness, within and across study countries. Modeled impacts are evaluated for young children and WRA in each country context in terms of achieving dietary adequacy, and in terms of child‐lives saved for three micronutrients (vitamin A, folic acid, and zinc). Costs include start‐up and operational costs faced by governments and industry, and premix costs, which are generally the main driver of fortification program costs [18]. Results are combined to provide estimates of the cost‐effectiveness of alternative premix choices in reducing dietary inadequacies and reducing child mortality in different country contexts. The results will be directly useful for the three countries included in this analysis, and the patterns of results will be useful in guiding bouillon program design discussions more broadly.

## 2 Methods and Data

### 2.1 Estimating the Nutritional Impacts of Bouillon Fortification

Estimates of the nutritional impact of bouillon fortification were based on modeling of household food consumption data. Specifically, we used household food consumption data from the 2018–2019 Enquête Harmonisée sur les Conditions de Vie des Ménages (EHCVM) in Burkina Faso and Senegal and the 2018–2019 Nigeria Living Standards Survey (NLSS) in Nigeria [30]. We matched each food in each country's respective food list with entries from the West African Food Composition Table [31], supplemented with entries from the Nutrition Coordinating Center Nutrient Database for Standard Reference [32] and the Malawian food composition table [33]. For micronutrients provided to those reached^2^ by existing LSFF programs in each country (fortified refined oil and wheat flour in Burkina Faso and Senegal and fortified refed oil, sugar, margarine, wheat flour, and maize flour in Nigeria), we accounted for the contribution of existing LSFF by increasing the micronutrient content of each fortifiable food by the estimated additional amount provided by LSFF based on current estimated compliance with industry standards (see Refs. [11−13] for specific existing LSFF program modeling assumptions). Then, combined with respondent recall of the quantity of each food consumed by members of the household in the past 7 days, we estimated total daily apparent household intake of energy, vitamin A, folate, vitamin B12, iron, and zinc.

We then converted estimates of total daily apparent household intake of each micronutrient to apparent nutrient density of the household diet^3^ by dividing the total daily apparent nutrient intake by total daily apparent energy intake, expressed per 1000 kcal [34]. To estimate dietary micronutrient adequacy, we compared the nutrient density of the household diet to the critical nutrient densities of children 6–59 months of age and WRA, where critical nutrient densities were calculated as the age‐ and sex‐specific estimated average requirement (EAR) divided by the age‐ and sex‐specific energy requirement, expressed in per 1000 kcal. The EARs for children and WRA were selected based on the household demographic information (age and sex) collected alongside the household food consumption data.

Vitamin A, folate, vitamin B12, and iron requirements were from the US Institute of Medicine, assuming 10% iron bioavailability [35, 36]. Zinc requirements were estimated first by using published equations [37, 38] to calculate fractional zinc absorption as the ratio of absorbed zinc to total zinc for each target household member (child and/or WRA). Then, we estimated critical zinc densities by adjusting physiological zinc requirements for children [39] and WRA [40] by the estimated person‐specific fractional zinc absorption and dividing by age‐ and sex‐specific energy requirements. We assessed the adequacy of the household diet plus existing LSFF for meeting vitamin A, folate, vitamin B12, and zinc requirements using the EAR cut‐point method, applied to the nutrient density estimates. To account for non‐normally distributed iron requirements among target household members, we assessed iron adequacy using the full probably method [22].

We estimated the marginal contribution of bouillon fortification to dietary adequacy by multiplying daily apparent bouillon consumption at the household level by each modeled bouillon fortification level and recalculating the nutrient density of the household diet.^4^ We then compared nutrient densities with bouillon fortification to critical nutrient densities. To recognize the possibility that compliance with bouillon fortification standards may be imperfect, we adjusted bouillon fortification levels to reflect the assumption that 75% of bouillon would be fortified to the specified level. We also assumed that, without the addition of compounds to enhance absorption, 2% of iron added to bouillon via fortification would be absorbed [41, 42]. For each micronutrient, we modeled a range of potential bouillon fortification levels: vitamin A (40−250 µg/g bouillon), folic acid (20−120 µg/g), vitamin B12 (0.2−2 µg/g), iron (0.6−5 mg/g), and zinc (0.6−5 mg/g). We also modeled an additional scenario in which bouillon fortification levels were selected to meet 30% of the Codex nutrient reference values (NRVs), calculated assuming 2.5 g of bouillon consumption per day among adults (96 µg vitamin A/g bouillon, 28.8 µg folic acid/g bouillon, 0.288 µg vitamin B12/g bouillon, 2.64 mg iron/g bouillon, and 1.68 mg zinc/g bouillon).

The effectiveness of bouillon fortification for improving dietary micronutrient adequacy was then calculated as the percentage and number of children or WRA effectively covered via bouillon fortification. Specifically, the percent of children or WRA effectively covered by each potential bouillon fortification scenario was calculated as the prevalence of inadequacy in the absence of bouillon fortification minus the prevalence of inadequacy with bouillon fortification. We converted the prevalence of children and WRA effectively covered to the number of children and WRA effectively covered by multiplying effective coverage (%) by the estimated total population of children 6–59 months or WRA in each year of the 10‐year time horizon [43]. Note that because we assumed a 2‐year startup period for bouillon fortification, effective coverage was set to 0 in years 1 and 2.

### 2.2 Using the Lives Saved Tool to Estimate Child‐Lives Saved

Estimates of the proportion of the target population with nutrient density of the household diet below the critical nutrient density (i.e., the affected fraction) and effective coverage (i.e., the proportion of the population that achieves nutrient density above the critical density via bouillon fortification) described above were used as inputs into the Lives Saved Tool (LiST) to generate estimates of child‐lives saved due to bouillon fortification with vitamin A, zinc, and/or folic acid. The methods used to link these estimates to the LiST model have been described elsewhere in greater detail [4]. A summary is provided below.

The LiST model is a deterministic model developed to estimate the effect of public health intervention programs on maternal and child mortality in LMICs (https://www.livessavedtool.org/). The structure of the LiST model has been described previously [44, 45]. Briefly, the LiST model estimates lives saved due to an intervention by multiplying the number of cause‐specific deaths that could be averted by a given intervention, the change in coverage of that intervention, the affected fraction (proportion of the population to which the intervention benefits are applied), and the effectiveness of the intervention on cause‐specific mortality.

Methods have been developed to adapt the LiST model to estimate the impacts of LSFF programs on child mortality [46]. In addition to the use of estimates of affected fraction and effective coverage in the LiST model, intervention program‐specific effectiveness estimates were changed to reflect the estimated impacts of achieving dietary adequacy on child mortality, rather than the effects of supplementation programs on child mortality, which is the default in the LiST model. Specifically, the vitamin A supplementation pathway was used to estimate lives saved among children 6–59 months of age. Default LiST parameters were replaced with country‐specific estimates of apparent vitamin A inadequacy (affected fraction) and effective coverage for each modeled level of bouillon fortification. Effectiveness estimates were replaced to reflect the modeled effects of achieving dietary adequacy of vitamin A.^5^ Similarly, the zinc supplementation pathway in LiST was used to estimate lives saved among children 12–59 months. Default LiST parameters were replaced with country‐specific estimates of apparent zinc inadequacy (affected fraction) and effective coverage for each modeled level of bouillon fortification. Effectiveness estimates for zinc supplementation were replaced to reflect the estimated effect of achieving zinc dietary adequacy [4].

The impacts on child‐lives saved due to folic acid fortification were estimated outside of LiST using a modification of the expanded LiST model #2 described elsewhere [47] and LiST estimates of the number of annual live births in each country. Similar to the calculations used in the LiST model, this approach used the annual number of neural tube defect (NTD) cases, multiplied by the change in effective coverage, multiplied by the effectiveness (impact of increased folic acid intake on the NTD cases) to generate estimates of the number of NTD cases averted and child‐lives saved attributable to NTD deaths [4].

From a policy perspective, it can be useful to examine the effects and the cost‐effectiveness of *combinations* of micronutrients. This is challenging to do in terms of micronutrient dietary adequacy because we lack value weights to reflect the relative importance of achieving adequacy in different micronutrients.^6^ In the context of child‐lives saved, however, LiST provides an implicit weighting system based on micronutrient‐specific marginal contributions to reductions in child mortality that can be used to “aggregate” the contributions of different micronutrients at different levels of fortification [4].

### 2.3 Estimating the Costs of Bouillon Fortification Programs

An ingredient‐ and activity‐based approach was used to estimate the economic costs^7^ of planning, designing, launching, and operating bouillon fortification programs in each country from a societal perspective [18, 48]. This approach focuses exclusively on the marginal costs of fortification for governments, industry, and consumers who, in the end, may pay all fortification program costs via increased retail prices for fortified foods and/or taxes. Several steps are involved in estimating program costs: (a) estimating the average annual consumption of bouillon cubes; (b) determining which micronutrients, and the amounts of each, to include in the premix; (c) assessing the prices of fortificants and then estimating the total cost of premix, including shipping, handling, and storage; (d) estimating the proportion of bouillon that is locally produced versus imported; (e) estimating the government start‐up and operational costs; and (f) estimating industry start‐up and operational costs. All of these costs are assessed on an annual time step and summed over the temporal extent of the model, which is 10 years.^8^ The complete set of results of cost analyses are provided in Vosti et al. [18], but Table 1 provides a summary of the costs and cost burdens associated with bouillon fortification programs in Senegal, Burkina Faso, and Nigeria that would deliver 30% of Codex NRV in 2.5 g of product.

**TABLE 1:** Summary estimates of bouillon fortification costs in Senegal, Burkina Faso, and Nigeria.

Summary cost measures	Senegal	Burkina Faso	Nigeria
Total costs ^a^			
Total 10‐year costs	$27,689,836	$20,770,738	$642,733,105
Total annual average cost (over 10 years)	$2,768,984	$2,077,074	$64,273,311
Total start‐up costs	$649,952	$647,595	$910,730
Total nonpremix operational costs (average annual cost over 8 years)	$1,013,971 ($126,746)	$863,669 ($107,959)	$31,207,812 ($3,900,977)
Total premix costs (annual average costs over 8 years)	$26,025,914 ($3,253,239)	$19,259,475 ($2,407,434)	$610,614,563 ($76,326,820)
Unit costs			
Cost per metric ton of fortified bouillon^b^	$319	$323	$333
Cost per bouillon consumer reached	$0.20	$0.13	$0.34
Cost shares (%)			
Government	4%	5%	0.3%
Industry	2%	2%	4.7%
Premix^c^ (only, as a % of total cost)	94%	93%	95%

^a^ The hypothetical bouillon fortification program envisioned for this costing exercise delivers bouillon fortified with five micronutrients (VA, folic acid, B12, iron, and zinc) at 30% of Codex nutrient reference value (NRV) for women of reproductive age, assuming a 2.5 g/day serving size. Iodine is also included in the fortified cube, but it is not included in cost calculations since it is assumed to be delivered by iodized salt, an ingredient not included in our cost model, which focuses only on marginal costs of fortification with other nutrients.

^b^ Slight differences across countries in cost per metric ton are attributable to differences in wage rates and import duties.

^c^ Ultimately, the bulk of premix costs will be paid by consumers, for both nationally produced and imported products.

*Source*: Adapted from Vosti et al. [18]. All monetary values are expressed in 2021 USD.

### 2.4 Linking Nutritional and Child Mortality Benefits to Bouillon Fortification Program Costs

Linking nutritional and child mortality benefits to bouillon fortification program costs requires temporal synchronization and spatial harmonization. Temporally, all of the estimates generated by both the nutritional benefits, child mortality, and cost models represent the same simulation time period (in this case, 2021–2030). In addition, the temporal flows of benefits are tied to the specific time periods during which such nutritional benefits are expected to flow, for example, no benefits are generated during the assumed 2‐year bouillon fortification program start‐up periods, although costs will be incurred during these 2 years. Spatially, for this analysis, all results are reported at the national level.^9^ Generating efficiency and effectiveness estimates also requires clarity regarding modeled fortification program effects. Cost‐efficiency was defined as the cost per person reached, where reach was calculated as the % of the target group that acquired/consumed bouillon within the survey data collection period.^10^ Cost‐effectiveness was defined according to two outcomes: effective coverage (i.e., the % or number of individuals who achieve dietary adequacy that are attributable to a given program) and child‐lives saved (i.e., the number of child deaths averted that are attributable to a given program).

## 3 Results

### 3.1 Baseline Levels of Micronutrient Inadequacies

The estimated prevalence of micronutrient inadequacies at baseline (i.e., considering intrinsic nutrient content of foods and current LSFF programs) varied substantially across countries and across micronutrients within countries. Table 2 reports baseline prevalence of inadequacies for vitamin A, vitamin B12, folate, iron, and zinc for young children and WRA. Due to differences in relative dietary requirements, the prevalence of inadequacy were higher for WRA than those of young children for folate, vitamin B12, and iron, and the opposite was true for zinc. Table 2 also reports estimated average daily consumption of bouillon, which also varied substantially across countries and across target beneficiary groups within countries. The prevalence of dietary inadequacy and rates of bouillon consumption serve as our baselines and have implications for the effectiveness and cost‐effectiveness of bouillon fortification programs.

**TABLE 2:** Baseline prevalence of micronutrient inadequacy and average bouillon consumption for young children and women of reproductive age (WRA, 15–49 years), by country.

	Senegal		Nigeria		Burkina Faso	
	Baseline micronutrient inadequacy^a^ among children (%)	Baseline micronutrient inadequacy^a^ among WRA (%)	Baseline micronutrient inadequacy^a^ among children (%)	Baseline micronutrient inadequacy^a^ among WRA (%)	Baseline micronutrient inadequacy^a^ among children (%)	Baseline micronutrient inadequacy^a^ among WRA (%)
Vitamin A	66%	66%	21%	20%	91%	93%
Vitamin B12	25%	29%	39%	42%	61%	66%
Folate	26%	42%	4%	14%	15%	34%
Iron	31%	43%	12%	24%	14%	23%
Zinc	74%	66%	39%	31%	44%	38%
Average apparent bouillon consumption among consumers^b^	0.9 g/child/day	1.9 g/WRA/day	1.9 g/child/day	3.7 g/WRA/day	0.5 g/child/day	1.1 g/WRA/day

^a^ Micronutrient inadequacy assessed at baseline (2018–2019, prior to introduction of fortified bouillon); this value included intrinsic dietary sources of micronutrients and those provided by existing fortification programs performing at current levels of adherence to standards, where “current” was estimated based on the most recent data available on average current fortification levels of existing LSFF programs in each country.

^b^ *Source*: From Adams et al. [11, 12, 13].

### 3.2 Cost‐Effectiveness of Bouillon Fortification in Reducing Micronutrient Inadequacies

**FIGURE 1:**
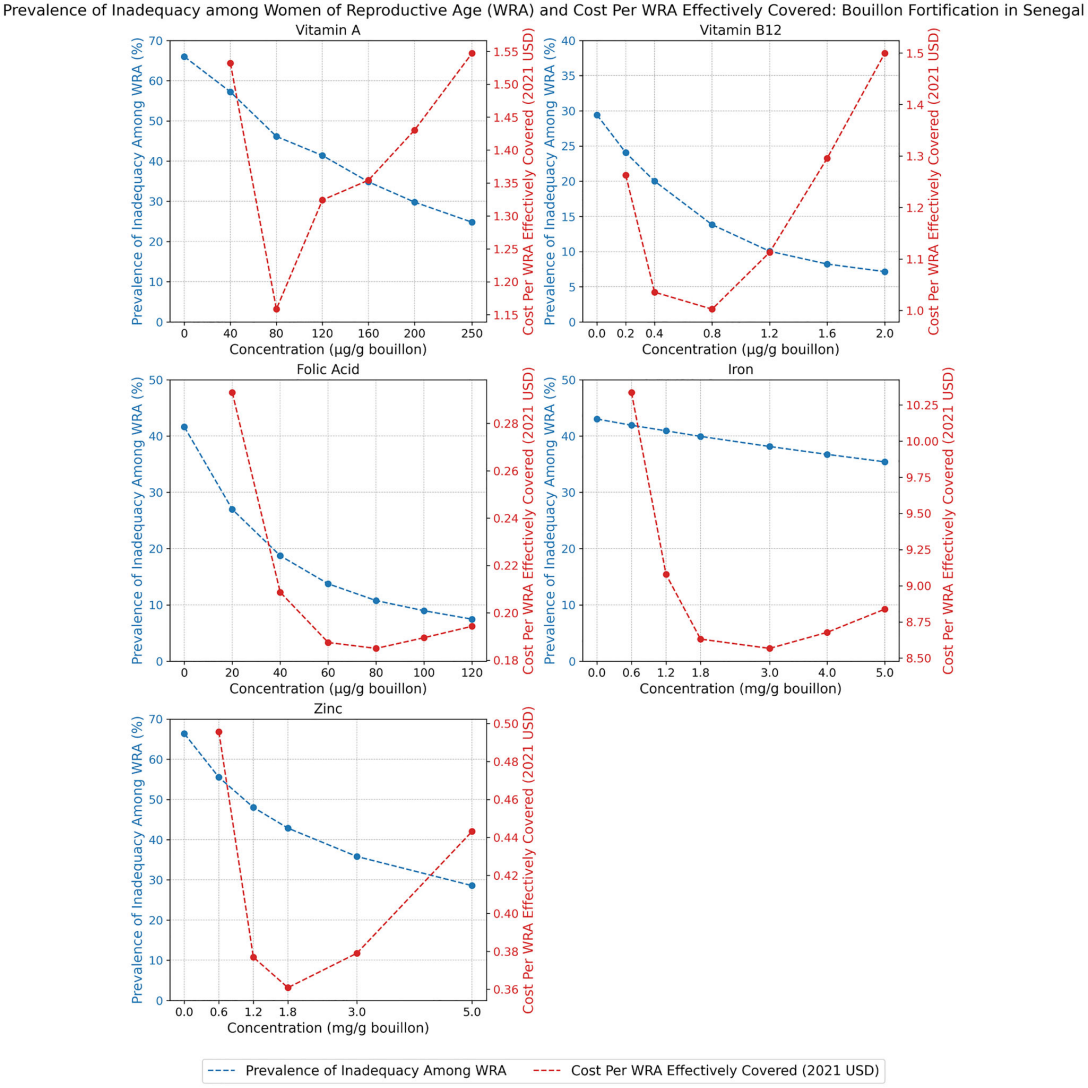
Prevalence of micronutrient inadequacy and cost per woman of reproductive age (WRA, aged 15–49) effectively covered under alternative bouillon fortification scenarios, WRA in Senegal.

**FIGURE 2:**
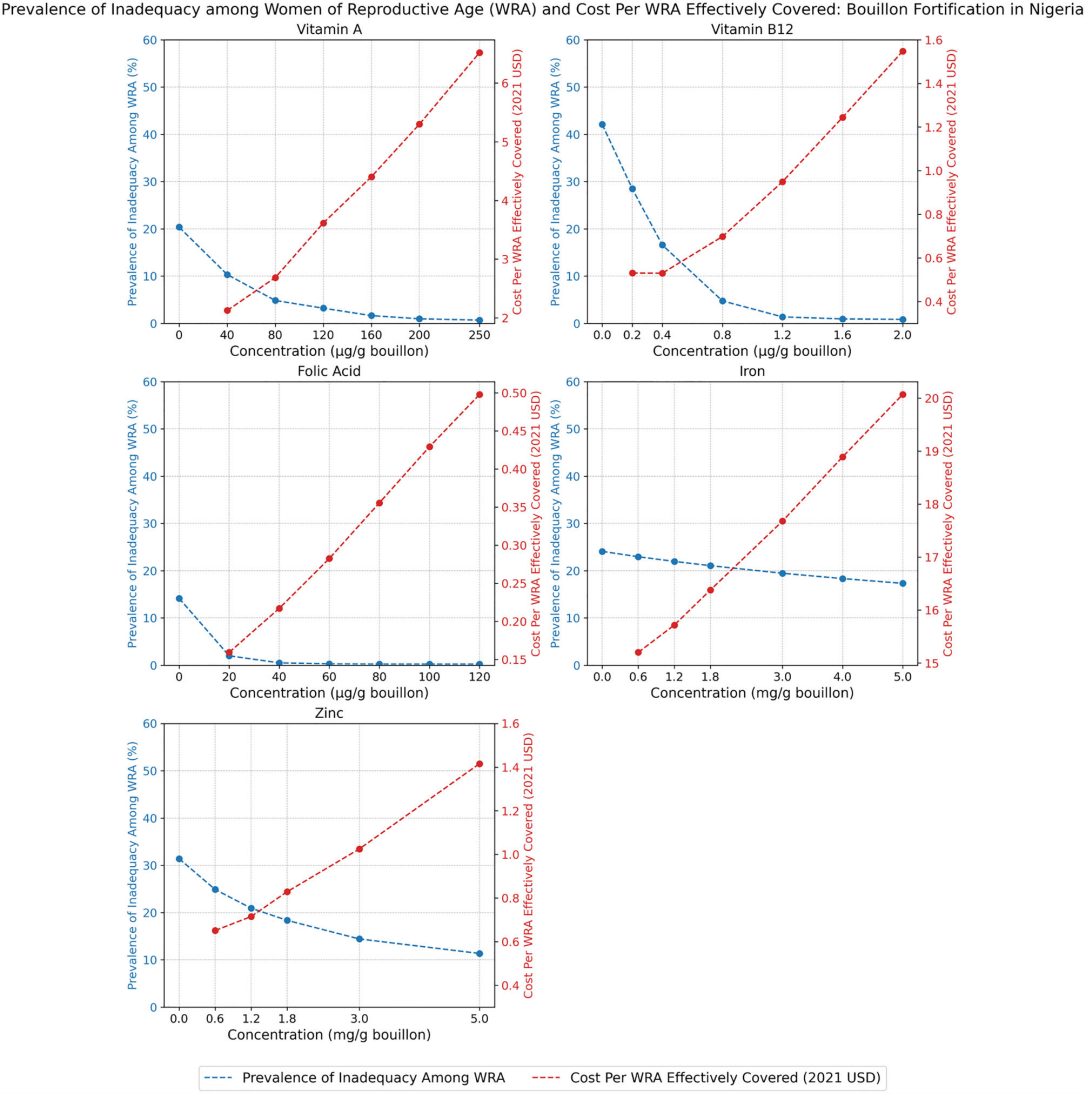
Prevalence of micronutrient inadequacy and cost per woman of reproductive age (WRA, aged 15–49) effectively covered under alternative bouillon fortification scenarios, WRA in Nigeria.

**FIGURE 3:**
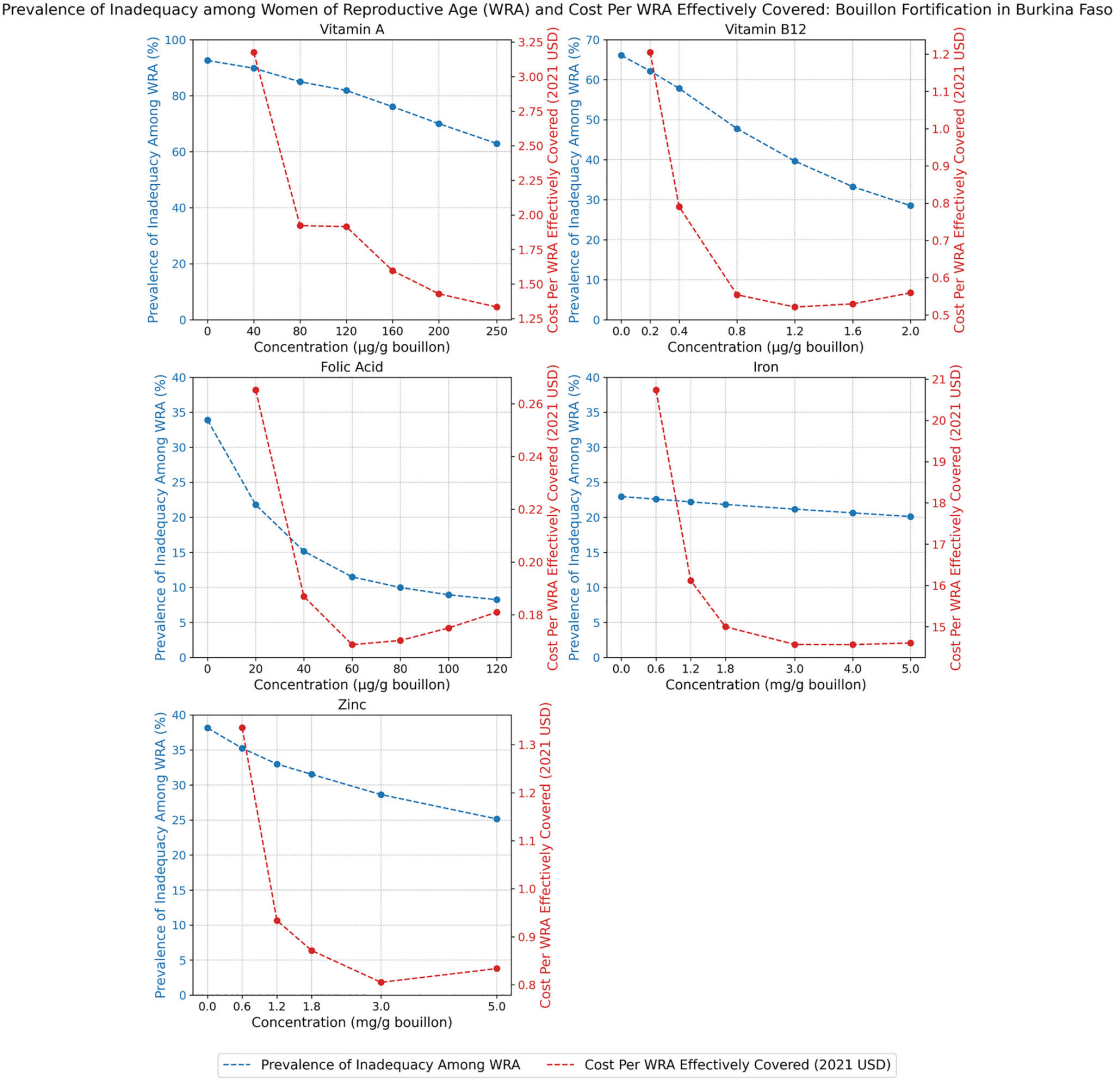
Prevalence of micronutrient inadequacy and cost per woman of reproductive age (WRA, aged 15–49) effectively covered under alternative bouillon fortification scenarios, WRA in Burkina Faso.

The impacts of alternative levels of bouillon fortification on the prevalence of micronutrient inadequacies for vitamin A, vitamin B12, folate, iron, and zinc, and on the cost per individual effectively covered for each of these micronutrients, are reported for WRA in Figure 1 for Senegal, Figure 2 for Nigeria, and Figure 3 for Burkina Faso.^11^

In general, one would expect downward‐sloping, curvilinear relationships between estimated prevalence of inadequacy and the micronutrient content of bouillon; with increasing doses, fewer cases of micronutrient inadequacy remain to be resolved. However, the slopes of these curves can vary depending on: (a) the size of dietary gaps (i.e., the difference between estimated nutrient intakes and nutrient requirements: if the gaps are small, they can be closed at lower fortification levels, but if they are larger, higher fortification levels are required to close them); (b) how much bouillon is being consumed; and (c) the estimated absorption of micronutrients consumed. Indeed, although the estimated prevalence of inadequacy based on diets and existing LSFF programs alone vary substantially by country and by socioeconomic groups within countries, the curvilinear patterns of reductions in prevalence of inadequacy as the amounts of micronutrients added to a fortified bouillon cube increase were similar across countries (see below). The same was true for patterns of reductions in child mortality for vitamin A, zinc, and folic acid (considered individually or jointly).

Since the bouillon fortification program operational costs were approximately linear in premix costs (and premix costs, which can be a substantial component of total ingredient costs, were approximately linear in levels of fortification) [18], combining concave benefit functions with linear cost functions, one would generally expect cost‐effectiveness curves to be U‐shaped relationships.

Indeed, the case of Senegal (Figure 1) depicts these expected relationships for each of the micronutrients examined, although the positions in space and the slopes vary by micronutrient, in part because the metrics along all three axes vary across micronutrients. For example, the impacts of increasing levels of vitamin B12 in bouillon reduced the prevalence of inadequacy from roughly 29% of WRA (based on diets and existing LSFF programs, alone) to roughly 7% at a fortification level of 2.0 µg vitamin B12 per gram of bouillon. At the other extreme, addition of iron to bouillon was predicted to generate relatively small reductions in the prevalence of dietary inadequacy in that micronutrient under the assumption of 2% absorption of iron from bouillon, for example, tripling the amount of iron added to bouillon (from 0.6 to 1.8 mg/g) reduced dietary inadequacy in iron by only approximately 2 percentage points. The cost per WRA effectively covered with vitamin B12 was roughly $1.25 at an initial dose of 0.2 µg/g of bouillon, then fell to approximately $1.00 at a fortification level of 0.8 µg/g of bouillon, and then rose to approximately $1.50 per WRA effectively covered at the highest modeled dose of 2.0 µg/g of bouillon.

Figure 2 depicts the predicted impacts in the case of Nigeria of increased levels of bouillon fortification for each of the five micronutrients included in this analysis on the prevalence of inadequacy and on cost‐effectiveness, again for the case of WRA. Note that the prevalence of inadequacy in vitamin B‐12, folate, and vitamin A were reduced to approximately zero at higher levels of bouillon fortification, with corresponding sharp increases in cost per WRA effectively covered. Once again, due to modeled limits on absorption, the impacts of fortification on inadequacies of zinc and iron were more muted; therefore, estimates of cost‐effectiveness rose sharply in both cases. That said, the cost‐effectiveness of zinc even at the *highest* level modeled (∼$1.42 per WRA effectively covered at 5.0 mg/g) was substantially lower than the cost‐effectiveness of the *lowest* level of iron fortification modeled (∼$15.0 per WRA effectively covered at 0.6 mg/g).

Finally, regarding the micronutrient‐specific nutritional benefits and cost‐effectiveness of alternative levels of bouillon fortificant, Figure 3 reports results for the case of Burkina Faso. As noted in Table 1, baseline dietary inadequacy of vitamin A and vitamin B12 were very high in Burkina Faso relative to those estimated for Senegal and Nigeria. High baseline levels of inadequacy generated benefit curves that are downward sloping; these combined with linear cost curves to generate generally downward sloping cost‐effectiveness curves, that is, cost‐effectiveness estimates fell almost continuously as levels of bouillon fortification increased, becoming slightly upward‐sloping in the cases of vitamin B12 and folic acid at higher levels of fortification.

### 3.3 Cost‐Effectiveness of Bouillon Fortification in Reducing Child Mortality

Viewed through the lens of child mortality attributable to vitamin A, zinc, and/or folate inadequacies (expressed in terms of the total number of child‐lives saved over the 8‐year operational phase of a bouillon fortification program) [4], patterns of effectiveness and cost‐effectiveness emerge across countries that are similar to those reported above for micronutrient inadequacies among WRA.^12^

**FIGURE 4:**
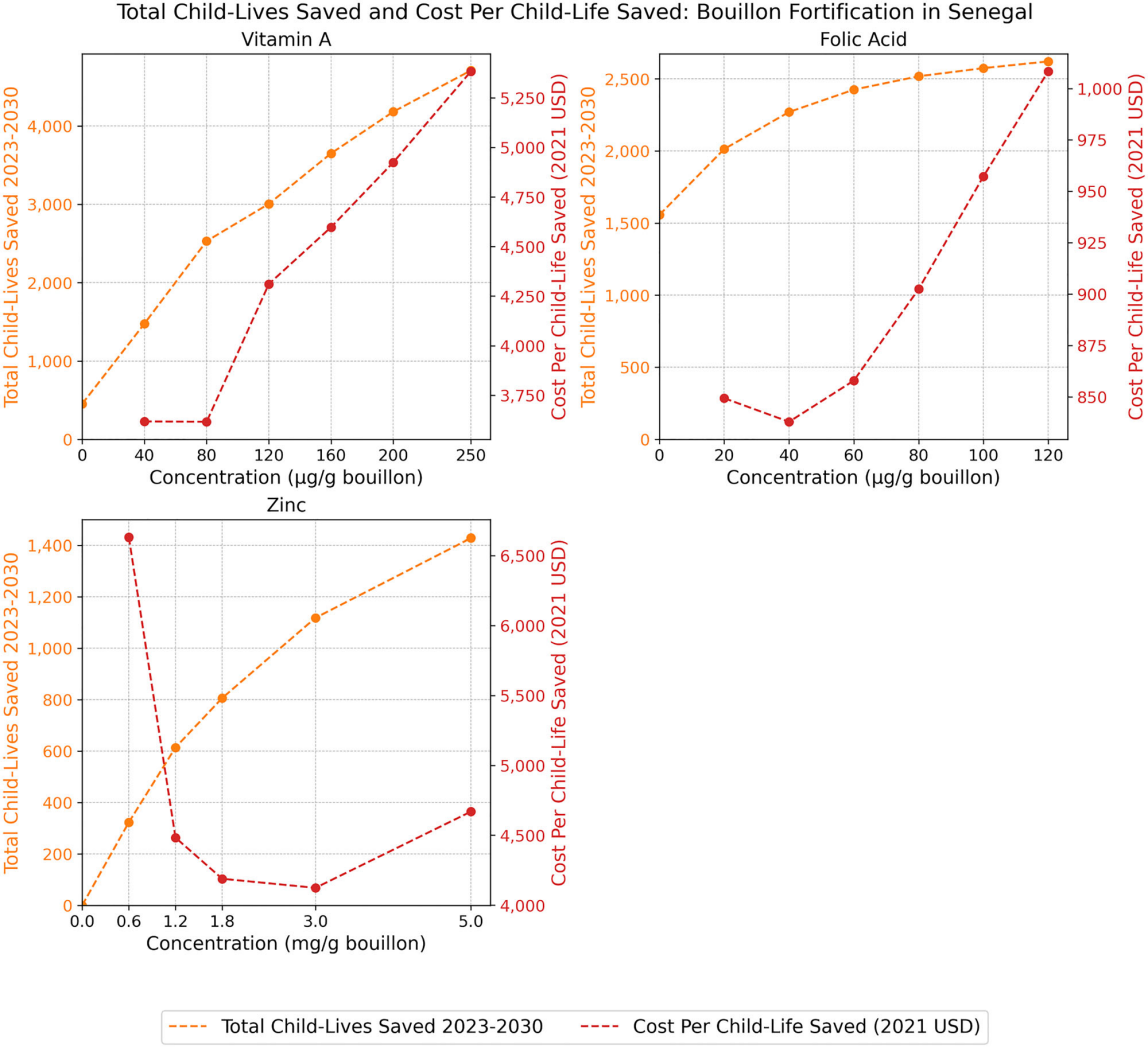
Total number of child‐lives saved 2023–2030 and cost per child‐life saved in Senegal. *Note*: Nonzero total lives saved estimates at zero levels of bouillon fortification are attributable to existing large‐scale food fortification (LSFF) programs.

In the case of Senegal (Figure 4), the marginal contributions to child‐lives saved for each of the micronutrients became smaller as the levels of bouillon fortification increased, that is, all of the total child‐lives saved curves were concave, since every life saved reduced the pool of lives that could be saved by higher levels of fortification. Related, the addition to bouillon at the lowest levels of nutrients modeled would lead to a substantial reduction in cost per child‐life saved for zinc and a moderate reduction for folic acid, but not for vitamin A. However, cost per child‐life saved rose as fortification levels increased, steeply so for vitamin A and folic acid.

**FIGURE 5:**
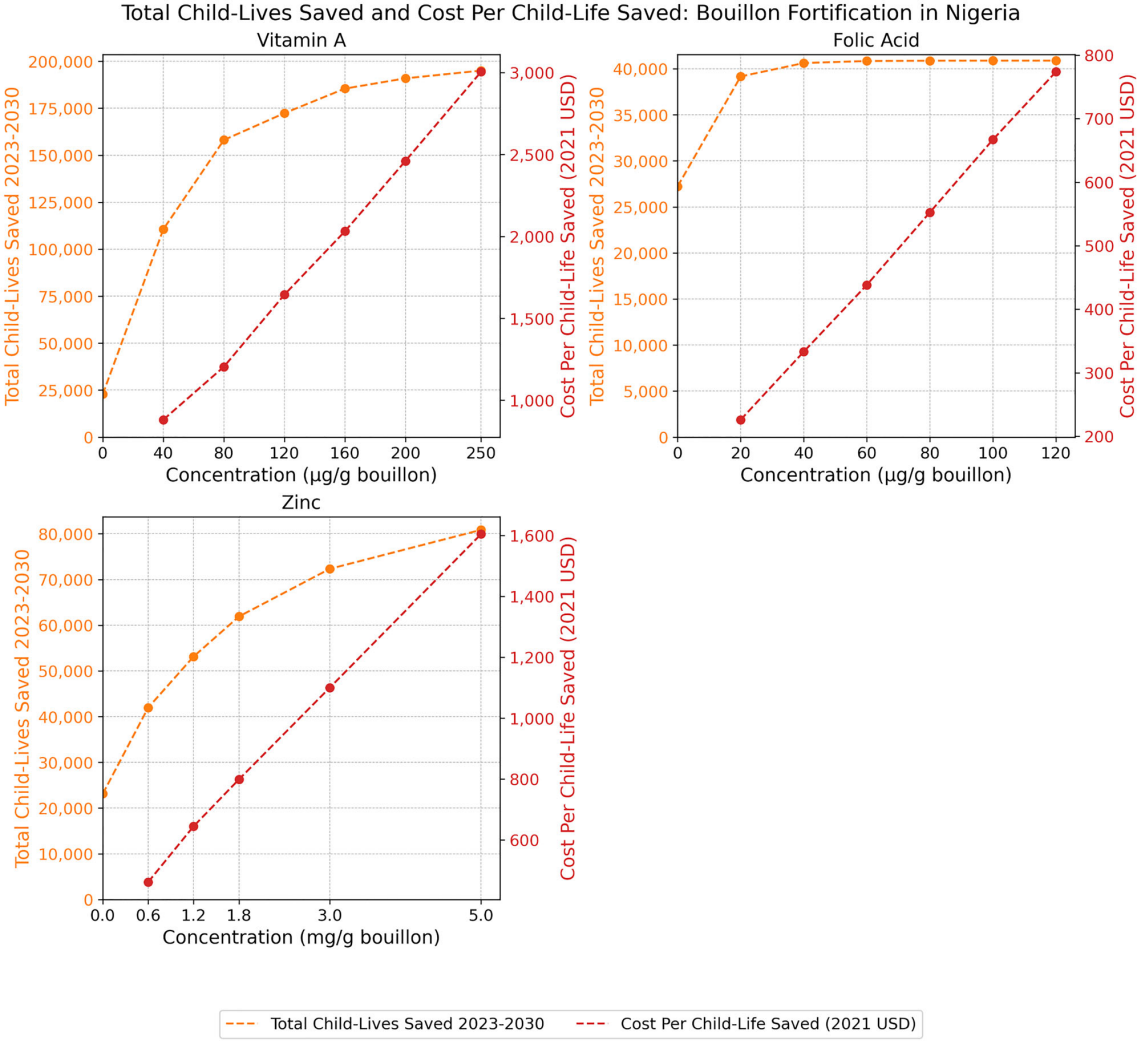
Total number of child‐lives saved 2023–2030 and cost per child‐life saved in Nigeria. *Note*: Nonzero total lives saved estimates at zero levels of bouillon fortification are attributable to existing large‐scale food fortification (LSFF) programs.

**FIGURE 6:**
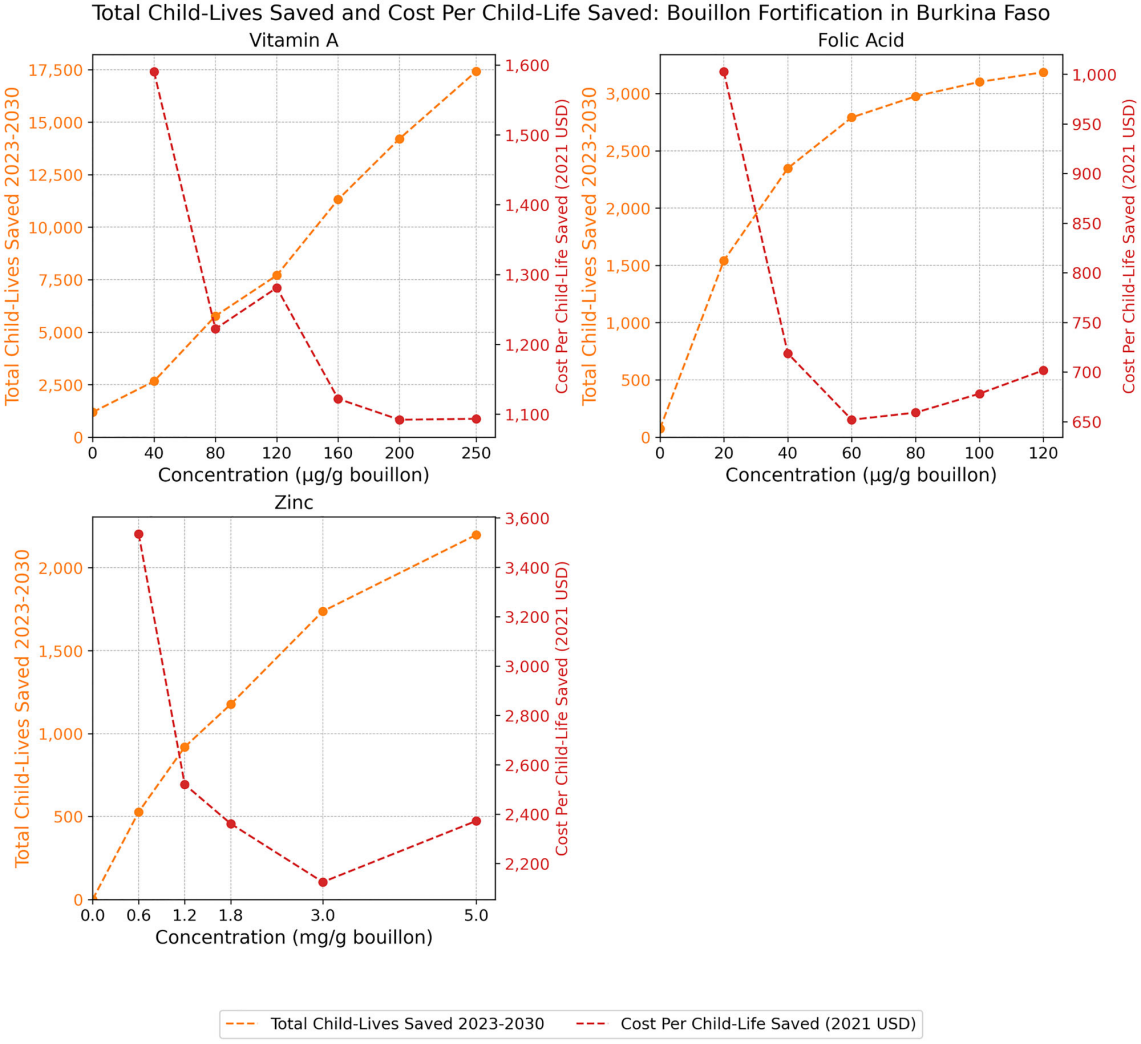
Total number of child‐lives saved 2023–2030 and cost per child‐life saved in Burkina Faso. *Note*: Nonzero total lives saved estimates at zero levels of bouillon fortification are attributable to existing large‐scale food fortification (LSFF) programs.

In Nigeria, the marginal reductions in child mortality associated with increased fortification levels for each of the micronutrients (Figure 5) declined more swiftly than those for Senegal (Figure 4) or Burkina Faso (Figure 6). Indeed, for folic acid, mortality reductions associated with fortification at levels higher than 40 µg/g of bouillon were practically zero as the prevalence of folate inadequacy was reduced to zero. These increasingly small contributions to mortality reduction as fortification levels increased led to sharply increasing costs per child‐life saved, for example, more than tripling for vitamin A from ∼$900 to ∼$3000 per child‐life saved.

Finally, for Burkinabe children, the proportional (to population size) marginal benefits of higher levels of vitamin A fortification were much higher than those reported for Senegal and Nigeria, and tended to remain so (Figure 6). These consistently large marginal benefits as fortification levels increased gave rise to steeply *decreasing* cost‐effectiveness curves. Similar patterns emerged for zinc (up to 3.0 mg/g) and folic acid (up to 60 µg/g); higher levels of fortification led to increases in cost per child‐life saved thereafter. Cost‐effectiveness for vitamin A remained at its lowest point (∼$1100 for child‐live saved) even at the upper bound of modeled fortification levels, indicating that the number of child‐lives saved was increasing in proportion to increases in premix costs.

**FIGURE 7:**
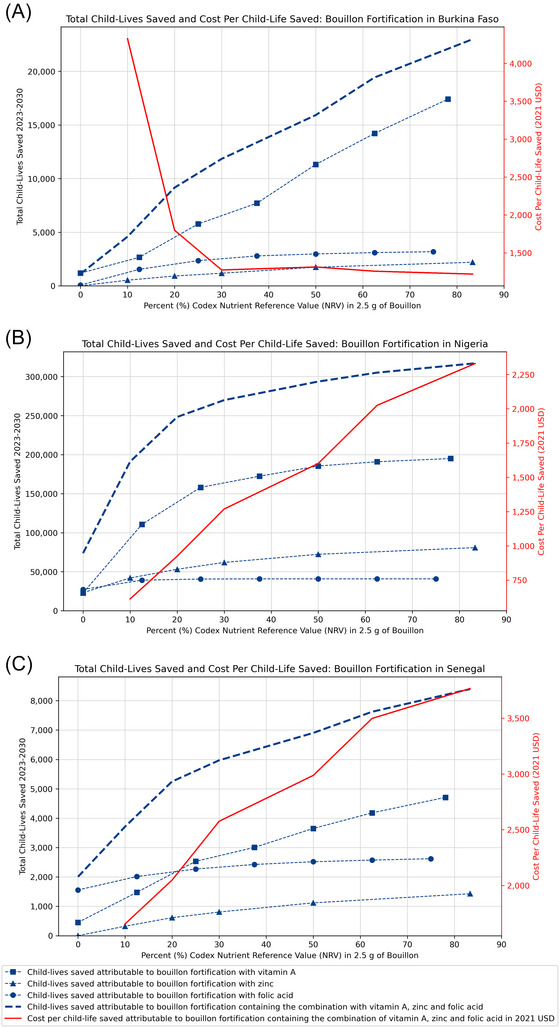
Total child‐lives saved 2023–2030 and cost per child‐life saved by level of bouillon fortification with vitamin A, folic acid, and zinc, individually and combined, according to the Codex nutrient reference values (NRVs).

Figure 7 reports the individual and joint effects of increasing levels of fortification in child‐lives saved in the three country contexts studied here. When comparing the sets of figures above with those below, it is important to note that the metrics along the horizontal axes are different from those used above; the previous set of three figures (Figure 4 through Figure 6) are expressed in terms of concentrations of micronutrient fortification (mcg/g or mg/g bouillon); in Figure 7, the results are expressed in terms of percent of Codex NRVs for WRA per 2.5 g of bouillon. The shapes and positions in space of micronutrient‐specific total child‐lives saved curves in the two sets of figures will match exactly, but the *composite* measure of the impacts on child‐lives saved involving all three micronutrients jointly is complicated by the fact that (say) 30% of Codex NRVs per serving represent different absolute amounts of each micronutrient.^13^

In Senegal (top of Figure 7), systematically increasing fortification levels in the context of percent of Codex NRVs per serving led to continued gains in child‐lives saved, but these gains began to taper off after reaching ∼20% of Codex NRVs per serving, which was approximately the point at which the marginal life‐saving benefits of folic acid were overtaken by those of vitamin A. Increasing all three micronutrients by the same percentage in NRV per serving (bold, dashed blue line) generated a strictly increasing cost‐effectiveness curve, from ∼$1500 to ∼$3700 per child‐life saved.

In the case of Nigeria (middle of Figure 7), the life‐saving benefits of all of the micronutrients dropped off quickly, relatively to those in other countries, which led to a “flattening” of the joint micronutrient benefit curve beginning at about 30% of Codex NRVs. This led to a roughly quadrupling of the cost per child‐life saved of the three‐micronutrient premix over the modeled levels of fortification.

Finally, results for the case of Burkina Faso (bottom of Figure 7) were quite different from those of the other country case studies. First, the marginal life‐saving impacts of additional amounts of vitamin A added to the premix remained constant over the entire range of modeled levels of fortification. Second, as a consequence, the cost per child‐life saved dropped significantly between 10% and 30% of Codex NRV in 2.5 g and essentially remained constant at ∼$1100 per child‐life saved for the entire remaining set of modeled fortification levels.

### 3.4 Details of Cost‐Effectiveness for a Bouillon Fortification Program Delivering 30% of Codex NRV

In what follows, we focus on a specific level of bouillon fortification (a premix that contains 30% of Codex NRV in 2.5 g of bouillon)^14^ and present more detailed information on the modeled cost and cost‐effectiveness of this formulation in terms of achieving micronutrient adequacy (Table 3, sequentially for Senegal, Nigeria, and Burkina Faso) and in terms of child‐lives saved (Table 4 for all three country case studies).^15^ The first three columns in each section of Table 3 report the cost, cost per capita, and cost per consumer for each of the micronutrients included in a bouillon fortification program delivering 30% of Codex NRV, and, in the final rows of each table, for the combination of the five micronutrients included in this study.^16^

**TABLE 3:** Bouillon 10‐year program costs, impacts, cost‐efficiency, and cost‐effectiveness for children and women of reproductive age (WRA).

Bouillon fortified with 30% Codex in 2.5 g for^a^:	Total program cost (000s USD)	Cost per capita^b^ (USD)	Cost per consumer reached^b^ (USD)	Cost per consuming child reached^b^ (USD)	Baseline inadequacy among children^c^ (%)	Bouillon's effective coverage^d^ (# of children)	Bouillon's effective coverage^e^ (% of children)	Cost per child effectively covered (USD)	Cost per consuming WRA reached^b^ (USD)	Baseline inadequacy among WRA^c^ (%)	Bouillon's effective coverage^d^ (# of WRA)	Bouillon's effective coverage^e^ (% of WRA)	Cost per WRA effectively covered (USD)
Senegal													
VA	$10,674	$0.07	$0.08	$0.57	66%	4,123,359	20%	$2.59	$0.30	66%	7,898,897	20%	$1.35
B12	$3222	$0.020	$0.023	$0.17	25%	1,290,039	6%	$2.50	$0.09	29%	2,830,759	7%	$1.14
Folic acid	$1789	$0.011	$0.013	$0.10	26%	2,427,257	12%	$0.74	$0.05	42%	7,429,890	19%	$0.24
Iron	$14,847	$0.09	$0.10	$0.80	31%	668,136	3%	$22.22	$0.42	43%	1,741,857	4%	$8.52
Zinc	$3257	$0.021	$0.023	$0.17	74%	3,328,624	16%	$0.98	$0.09	66%	8,891,267	22%	$0.37
All five MNs	$27,690	$0.17	$0.20	$1.48	n/a^f^	n/a	n/a	n/a	$0.78	n/a^f^	n/a	n/a	n/a
Nigeria													
VA	$227,661	$0.117	$0.119	$0.77	21%	47,987,485	16%	$4.74	$0.51	20%	70,700,421	15%	$3.22
B12	$45,773	$0.024	$0.024	$0.15	39%	61,640,459	20%	$0.74	$0.10	42%	89,241,387	20%	$0.51
Folic acid	$10,780	$0.006	$0.006	$0.04	4%	12,890,322	4%	$0.84	$0.02	14%	60,147,585	13%	$0.18
Iron	$329,304	$0.170	$0.173	$1.11	12%	10,381,188	3%	$31.72	$0.73	24%	19,090,666	4%	$17.25
Zinc	$46,570	$0.024	$0.024	$0.16	39%	36,631,796	12%	$1.27	$0.10	31%	58,095,088	13%	$0.80
All five MNs	$642,733	$0.332	$0.337	$2.16	n/a^f^	n/a	n/a	n/a	$1.43	n/a	n/a	n/a	n/a
Burkina Faso													
VA	$8179	$0.04	$0.05	$0.35	91%	2,480,640	9%	$3.30	$0.21	93%	3,667,234	8%	$2.23
B12	$2665	$0.01	$0.02	$0.11	61%	1,938,227	7%	$1.37	$0.07	66%	2,746,907	6%	$0.97
Folic acid	$1604	$0.008	$0.01	$0.07	15%	1,926,344	7%	$0.83	$0.04	34%	7,452,515	15%	$0.22
Iron	$11,267	$0.06	$0.07	$0.48	14%	326,659	1%	$34.49	$0.28	23%	772,068	2%	$14.59
Zinc	$2690	$0.01	$0.02	$0.12	44%	1,544,989	5%	$1.74	$0.07	38%	3,070,478	6%	$0.88
All five MNs	$20,771	$0.10	$0.13	$0.89	n/a^f^	n/a	n/a	n/a	$0.52	n/a	n/a	n/a	n/a

*Note*: All costs are reported in 2021 US dollars (USD).

^a^ VA (96 µg/g) of retinyl palmitate 250,000 IU/g (dry); B12, 0.288 µg/g of vitamin B12 0.1% WS; Folic acid, 28.8 µg/g of folic acid; Iron (2.64 mg/g) of micronized ferric pyrophosphate; Zinc, 1.68 mg/g of zinc oxide. For fortificant absorption parameters, see Adams et al. [11, 12, 13].

^b^ For these calculations, the denominators are: (i) total population; (ii) total population multiplied by reach; (iii) population of children 6–59 months multiplied by reach, among households that had at least one child 6–59 months of age in the survey data; and (iv) population of WRA multiplied by reach, among households that had at least one WRA in the survey data. Reach was defined as households that reported consuming any bouillon in the 7‐day period prior to the interview. Total program costs remain unchanged in all cost‐efficiency and cost‐effectiveness calculations.

^c^ Micronutrient inadequacy assessed at baseline (2018–2019, prior to the hypothetical introduction of fortified bouillon); this value includes natural dietary sources of micronutrients and those provided by existing fortification programs performing at current levels of adherence to standards, where “current” is estimated based on the most recent data available on average current fortification levels of refined oil and wheat flour in Burkina Faso.

^d^ Changes in the number of cases of dietary micronutrient inadequacy attributable to fortified bouillon, considering baseline diets and existing fortification programs.

^e^ Percentage of micronutrient inadequate children or WRA who achieve adequacy due to bouillon fortification.

^f^ n/a, not applicable.

**TABLE 4:** Bouillon 10‐year program costs, child‐lives saved, and cost‐effectiveness in Burkina Faso, Nigeria, and Senegal.

	Burkina Faso			Nigeria			Senegal		
Bouillon fortified with 30% Codex in 2.5 g for^a^:	Total program cost^d^ (000s USD)	Child‐lives saved^e^ (# of children)	Cost per child‐life saved^d^ (USD)	Total program cost (000s USD)	Child‐lives saved^e^ (# of children)	Cost per child‐life saved^d^ (USD)	Total program cost (000s USD)	Child‐lives saved^e^ (# of children)	Cost per child‐life saved^d^ (USD)
VA (96 µg/g)^b^	$8179	4536	$1803	$227,661	136,022	$1674	$10,674	2137	$4995
Folic acid (28.8 µg/g)^c^	$1604	1973	$813	$10,780	38,271	$282	$1789	1314	$1361
Zinc (1.68 mg/g)^b^	$2690	1112	$2419	$46,570	40,997	$1136	$3257	498	$6540
Combined VA, zinc, and folic acid	$9656	7590	$1272	$276,334	212,970	$1298	$12,670	3918	$3234

*Note*: All costs are reported in 2021 US dollars (USD).

^a^ VA, retinyl palmitate 250,000 IU/g (dry); Folic acid, folic acid; Zinc, Zinc oxide. For fortificant absorption parameters, see Adams et al. (2024).

^b^ Pathway in Lives Saved Tool (LiST) for VA saves lives among children 6–59 months of age; zinc pathway in LiST saves lives among children 12–59 months.

^c^ Pathway for folic acid is via women of reproductive age (WRA).

^d^ Bouillon fortification programs incur costs in each year of the 10‐year simulation period, but generate nutritional benefits in years 3–10.

^e^ Estimates are the marginal benefits of bouillon fortification, accounting for baseline diets and existing fortification programs.

In the top section of Table 3, we see that in the context of Senegal including some micronutrients will be substantially more expensive than others, for example, iron is the most expensive (10‐year total cost of ∼$14.8m)^17^ and folic acid is the least expensive (10‐year total cost of ∼$1.8m); fortifying with all micronutrients will cost ∼$27.7m over 10 years. Because bouillon is so widely consumed, the cost per capita and cost per consumer reached are similar, for example, for a program delivering all five micronutrients, shifting focus from the entire population to the bouillon‐consuming population increases average cost from ∼$0.17 to ∼$0.20.

The second set of columns in the top section of Table 3 focuses more narrowly on the population of children 6–59 months of age in Senegal. Note that the cost per consuming child reached is substantially higher than the cost per consumer reached (the 6‐ to 59‐month‐old population is only a fraction of the total population). The prevalence of micronutrient inadequacy varies by micronutrient (from 25% for vitamin B12 to 74% for zinc), as do the contributions of fortified bouillon to reducing micronutrient inadequacy (from 3% for iron to 20% for vitamin A), so the cost per child effectively covered ranges from $0.74 for folic acid to $22.22 for iron.

The final set of columns of the top section of Table 3 focuses on WRA in the context of Senegal. Essentially, the same patterns played out for WRA as was the case for young children, with some differences in levels of baseline dietary inadequacies and levels of effective coverage. For specific micronutrients, the cost per WRA reached varied between ∼$0.05 for folic acid and ∼$0.42 for iron. Levels of baseline inadequacies (once again, based on dietary intake plus the micronutrient contributions of existing LSFF programs) also varied across micronutrients, as did the impacts on dietary adequacy, for example, an iron fortification program would reduce inadequacy among WRA by ∼4 percentage points, while a zinc fortification program would reduce inadequacy by ∼22 percentage points. In terms of micronutrient‐specific cost‐effectiveness (cost per WRA effectively covered), the impact and cost results suggested a wide range of efficiency, from a low of ∼$8.52 for an iron fortification program to a high of ∼$0.24 for a folic acid program. Differences in cost‐effectiveness measures for WRA vis‐à‐vis those for children were attributable mainly to the relative size of the WRA population.

In terms of absolute fortification program costs (middle section of Table 3), the results for Nigeria (∼$643m over 10 years for all five micronutrients) were much higher than those reported for Senegal (∼$27.7m over 10 years for a premix including all five micronutrients); differences were primarily attributable to the quantity of bouillon in the food system to be fortified, which was a function of the size of the bouillon‐consuming population (218m vs. 16m for Nigeria and Senegal, respectively) and bouillon consumption patterns (3.7 vs. 1.9 g/WRA/day in Nigeria and Senegal, respectively). The normalized patterns of impacts, cost‐efficiency, and cost‐effectiveness were similar to those in Senegal. For example, in terms of cost per consumer reached, micronutrient‐specific estimates ranged from ∼$0.006 for folic acid to ∼$0.173 for iron; cost per consumer reached for a program containing all five micronutrients was ∼$0.337. The prevalence of micronutrient inadequacy varied by micronutrient (from 4% for folate and 39% for vitamin B12 and zinc), as did fortified bouillon's contribution to reducing micronutrient inadequacy (from 3% for iron to 20% for vitamin B12), so the cost per child effectively covered ranged from $0.74 for vitamin B12 to $31.72 for iron. Cost per bouillon‐consuming WRA reached remained highest for an iron fortification program (∼$0.73) and lowest for a folic acid fortification program (∼$0.02). Once again, micronutrient‐specific program costs and impacts combined to produce a similar but much wider pattern of results for cost‐effectiveness, ranging from ∼$0.18 per WRA effectively covered for a folic acid fortification program to ∼$17.25 per WRA effectively covered for an iron fortification program.

The results for the case of Burkina Faso are reported in the bottom section of Table 3. Total 10‐year total program costs were lower than those for Senegal, mainly because of differences in population size and also because of bouillon consumption patterns (average bouillon consumption in Burkina Faso was 1.1 vs. 1.9 g/WRA/day for Senegal); the latter also had implications for program impacts. Once again, similar patterns in normalized efficiency measures arose; cost per consumer reached was highest for the stand‐alone iron fortification program (∼$0.07) and lowest for the folic acid fortification program (∼$0.01). The prevalence of micronutrient inadequacy varied by micronutrient (from 14% for iron to 91% for vitamin A), as did fortified bouillon's contribution to reducing micronutrient inadequacy (from 1% for iron to 9% for vitamin A), so the cost per child effectively covered ranged from $0.83 for folic acid to $34.49 for iron. Similar patterns emerged for WRA; cost per bouillon‐consuming WRA reached and per WRA effectively covered were highest for the iron fortification program (∼$0.28 and ∼$14.59, respectively) and lowest for a folic acid fortification program (∼$0.04 and ∼$0.22, respectively).

Finally, in terms of results for a specific bouillon fortification program aiming to deliver 30% of Codex NRV in 2.5 g, Table 4 reports child‐lives saved modeled results for the three micronutrients (vitamin A, folic acid, and zinc) with links to child mortality, for each of the case study countries. For each country, we report the total 10‐year costs of a bouillon fortification program delivering the prescribed amounts of each of these micronutrients, the estimated number of child‐lives that would be saved by such a fortification program, and the cost per child‐life saved for each program (and for a program delivering all three micronutrients). Note that the sum total of the three single‐micronutrient fortification program costs was larger than that of the combined three‐micronutrient fortification program; lower costs for the combined programs were attributable mainly to start‐up costs, which were the same for the single‐micronutrient and the combined‐micronutrient programs.

Total program costs and the absolute number of child‐lives saved were orders of magnitude larger for Nigeria than for the other two countries; once again, this is attributable mainly to differences in population size. The cost‐effectiveness measures are more similar, with cost per child‐life saved via the combination of vitamin A, zinc, and folic acid‐fortified bouillon being lowest in Burkina Faso (∼$1272) and highest in Senegal (∼$3234). A key factor driving differences in cost‐effectiveness across countries was the relative size of the pool of micronutrient‐attributable deaths, which was in part determined by relative differences in dietary micronutrient inadequacies among children in the absence of bouillon fortification, as well as differences in the size of the child population in each country, which was lowest in Senegal and highest in Nigeria.

Since there is uncertainty in estimating program costs and the relationships between dietary adequacy and mortality, sensitivity analyses were undertaken using alternative estimates of program costs and alternative effectiveness estimates on child mortality for vitamin A, zinc, and folic acid. Ranges of estimates of (e.g.) the cost per child‐live saved were substantial. In the context of Senegal, for example, our preferred estimate is $3208 per child‐life saved; under a low‐cost/high‐impact scenario, this estimate falls to $2391, but under a high‐cost/low‐impact scenario, this estimate rises to $7278 per child‐life saved. A complete set of sensitivity analyses results for each country are reported in the Supplementary Material.^18^

### 3.5 Using the Results of Cost‐Effectiveness Analyses to Address a Concrete Policy Discussion: Evidence Used to Support Premix Formula Discussions in Nigeria

**FIGURE 8:**
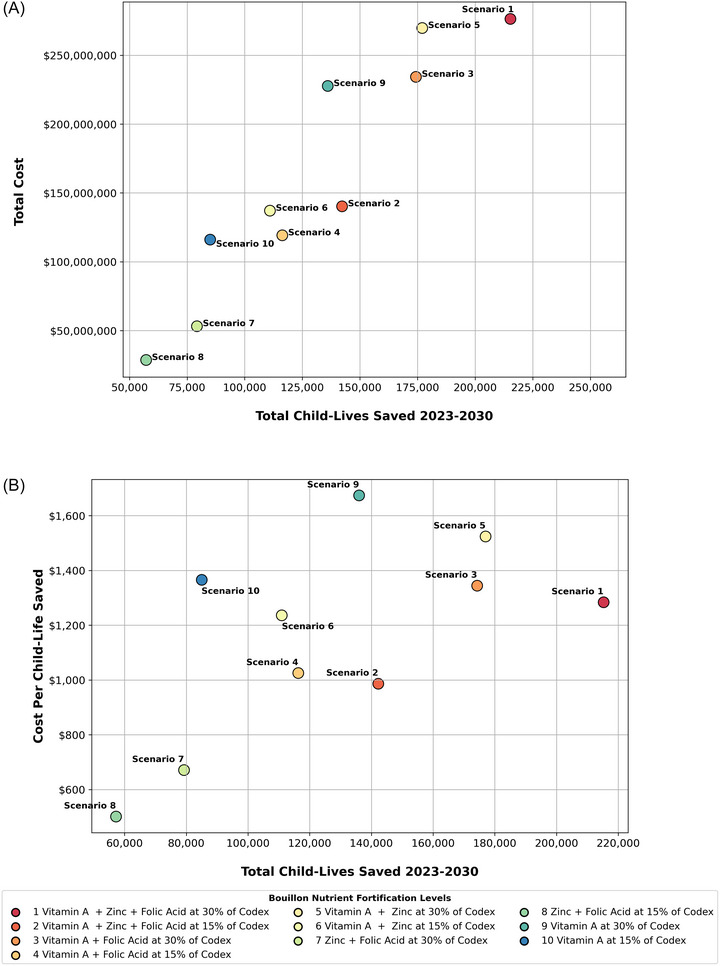
Total child‐lives saved and (A) total bouillon fortification costs and (B) cost per child‐live saved, by fortification scenario in Nigeria. All costs are presented in 2021 US dollars; fortification levels are expressed in percentages of Codex nutrient reference values (NRVs) in 2.5 g of bouillon; results are for a 10‐year simulation period, with 2‐year start‐up periods during which no mortality benefits accrue.

The perennial challenge in supporting policy discussions is to identify the bits of evidence that are relevant and convincing to stakeholders, and to tailor evidence delivery packages for easy and quick absorption [49]. In the context of bouillon fortification policy discussions in Nigeria, the research team presented very large amounts of modeled evidence in many different ways. One major challenge was to summarize this body of modeled evidence regarding the costs, impacts, and cost‐effectiveness for a small, agreed‐upon (by all stakeholders) set of alternative bouillon fortification programs. Our choices of levels of fortification were guided by Codex rules related to labeling [50]; our choices of micronutrients were restricted to the five included in this study and further restricted (in results presented below) to three micronutrients for which established methods exist to model the impact on child mortality (vitamin A, zinc, and folic acid). We chose child‐lives saved as our impact metric; all stakeholders could easily understand this metric and were supportive of it as a policy objective. The 10 combinations of alternative premix formulations that were considered by stakeholders in Nigeria are reported as *Scenarios* in Figure 8; the Scenarios are defined in the legend at the bottom of the figure.

The results reported in Figure 8 (top) focus on the total 10‐year program costs and the absolute numbers of child‐lives estimated to be saved in the 8 operational years of alternative bouillon fortification programs.^19^ *Scenario 1* (delivering 30% of Codex NRVs for the three micronutrients with pathways in LiST) was the most impactful and also the most expensive. *Scenario 5* and *Scenario 3* were next in line in terms of impact, but *Scenario 3* was substantially less expensive; *Scenario 9* was roughly as costly as *Scenario 3* but was much less impactful. The next cluster of alternative programs in terms of overall cost (and hence affordability) were *Scenarios 2*, *6*, *4*, and *10*; in this cluster, *Scenario 2* stands out as the most impactful. Finally, in terms of overall cost, *Scenarios 7* and *8* were the least expensive and among the lowest in terms of impact; note that neither of these scenarios contained vitamin A.

The final input into policy discussions in Nigeria dealt with the issue of efficiency or cost‐effectiveness of alternative bouillon fortification programs. Figure 8 (bottom) reports these results. Note that the two most cost‐effective programs (*Scenario 7* and *Scenario 8*) were those saving the fewest child‐lives because neither program delivered any vitamin A. The least cost‐effective program (*Scenario 9*) delivered only vitamin A at 30% of Codex NRV. Delivering only 15% of Codex NRV for vitamin A (*Scenario 10*) was more cost‐effective, but fewer child‐lives were saved. *Scenarios 1*, *3*, and *5*, which delivered combinations of life‐saving micronutrients at 30% of Codex NRV, were less cost‐effective than most other scenarios, but saved the largest numbers of child‐lives. *Scenarios 2*, *4*, *6*, and *10* all delivered 15% of Codex NRVs of vitamin A and one or more of the other life‐saving micronutrients. The range of cost‐effectiveness for this subset of bouillon fortification options (from ∼$550 to ∼$1700 per child‐life saved) was substantially lower than some estimates in the literature (e.g., $12,764 per child‐life saved by wheat flour fortified with iron and folic acid [51], ∼$10,000 per death averted by addressing micronutrient deficiencies generally [29]), but higher than other estimates (e.g., $211.70 per child‐life saved [2021 US$] by vitamin A‐fortified bouillon in Cameroon [10]).

Concrete policy discussions made simultaneous use of the evidence presented in Figure 8. For example, technical and commercial viability issues aside (and these were very important issues that could eliminate some scenarios from consideration), if policymakers could afford *Scenario 5*, then it would be worth spending a bit more to achieve the additional benefits offered by *Scenario 1*.^20^ The same was true when comparing *Scenarios 9* and *3*. In the total cost range of ∼$120m to ∼$145m, *Scenario 2* was the clear choice. Once again, *Scenarios 7* and *8* were the least expensive, but saved the fewest child‐lives.

## 4 Discussion

There is general agreement that cost is a key issue to consider when designing, managing, and evaluating LSFF programs [52, 53] and increasing awareness of the importance of the allocation of costs among stakeholder groups in determining the efficiency and sustainability of these programs [18]. However, less attention has been paid to the impacts of key LSFF program design characteristics on the impacts and cost‐effectiveness of LSFF programs. In particular, little is known about the effects of changes in the types and/or amounts of micronutrients added to fortifiable foods and condiments on program impacts and program costs. This paper breaks new ground by combining the modeled evidence on the impacts and costs of alternative fortified bouillon premix choices for five micronutrients (vitamin A, vitamin B12, folic acid, iron, and zinc) in three country contexts (Burkina Faso, Nigeria, and Senegal).

More specifically, we make the following contributions to the literature. First, this paper focuses on a novel delivery vehicle with very extensive geographical and socioeconomic reach, and hence broad potential to address micronutrient inadequacies and (for some micronutrients) child mortality. Second, we model an array of alternative combinations and levels of micronutrient fortification and assess the impacts, costs, and cost‐effectiveness along a continuum from low to higher levels of fortification. Third, doing so allows us to identify patterns of modeled efficiency in terms of achieving nutritional adequacy and child‐lives saved associated with alternative premix formulas within and across case study countries. Fourth, we discuss how these results can be used in choosing the types and amounts of fortificants to be considered when designing bouillon fortification programs.

Regarding policy guidance, cross‐site comparisons of the ranges of modeled cost‐effectiveness (from lowest to highest modeled levels of micronutrient concentrations) of the five micronutrients examined in terms of achieving nutritional adequacy among WRA identified clear patterns. In all cases and regardless of levels of fortificant concentrations, folic acid fortification was the most cost‐effective (owing to the relatively low price of folic acid), and iron fortification was the least cost‐effective (due to low absorption and the relatively high price of micronized ferrous pyrophosphate^21^). Vitamin A fortification was the second least‐cost‐effective fortificant (due to the relatively high price of retinol palmitate). Vitamin B12 and zinc occupied the second and third ranks in terms of efficiency, depending on the country context.

The shapes and positions in space of the benefit and cost‐effectiveness curves will depend on baseline prevalence of inadequacy, levels and amounts of bouillon consumption, and (by our design) the amounts of different micronutrients added to bouillon. Given the generally nonlinear nutritional and child‐lives‐saved benefit functions and the generally linear premix cost functions, one would expect U‐shaped cost‐effectiveness curves. The case of Senegal generated the expected U‐shaped nature of cost‐effectiveness curves, while the cases of Nigeria and Burkina Faso more generally characterized the “ends” and “beginnings” of these U‐shaped curves, respectively. Knowing the shapes of benefit curves can help identify the amounts of different fortificants to be included in premixes. For example, in the context of Nigeria, at relatively low levels of folic acid concentration, the modeled contributions of *additional* units of folic acid to reducing nutritional inadequacy among WRA were practically zero. The situation was similar in Nigeria for vitamin A and vitamin B12, though the levels of concentration at which marginal benefits disappeared were somewhat higher than in the case of folic acid. In each case, policymakers should generally avoid recommending concentration levels above those at which marginal benefits are low, even if the fortificants are very inexpensive.

One perennial challenge in supporting policy discussions is to identify elements of the overall evidence base that are relevant and convincing to stakeholders, and to tailor, package, and deliver *that* evidence for easy and quick absorption.^22^ For bouillon fortification policy discussions, the research team summarized the modeled evidence regarding the costs, impacts, and cost‐effectiveness for a small, agreed‐upon set of alternative bouillon premixes. Our choices of levels of fortification were guided by Codex rules related to labeling [50]; our choices of micronutrients were restricted to the five included in this study. For some presentational materials, evidence was further restricted to vitamin A, zinc, and folic acid, for which established methods exist to model the impact on child mortality, a program impact metric that all stakeholders could easily understand and support as a policy objective.

The results presented here have several limitations. First, while estimated dietary intake patterns vary substantially within and across the three countries chosen for analysis (Burkina Faso, Nigeria, and Senegal), modeled results presented here might not reflect the realities in other contexts, even within West Africa. Second, we focus on five micronutrients (and selected fortificants for each of them); the analysis would be enhanced by including a broader array of micronutrients of public health concern (e.g., additional B vitamins) and of fortificants (especially in the case of iron). Third, some presentational materials tended to focus on the levels of micronutrient concentrations associated with 15% and 30% of Codex NRV in 2.5 g of product; for some micronutrients, levels of fortification well above those cut‐offs might be both technically and commercially feasible. Fourth, modeled impact results are based on household‐level data; assumptions (set out in the Methodology section) were made to disaggregate to the levels of target beneficiary groups within households (namely, for this analysis, young children and WRA). Alterations of these key assumptions might influence the results and the policy implications derived from them. Fifth, and related, the paper focused exclusively on young children and WRA; other consumers of fortified bouillon (e.g., adolescent girls and men) might also benefit, but these benefits are not included in our analyses. Finally, this paper did not address the technical, commercial, or other challenges associated with developing and marketing commercially viable multifortified bouillon cubes. Technical or other constraints, once known, could be introduced into this analysis; doing so may reduce the combinations and ranges of amounts of some fortificants to be considered in designing bouillon fortification programs, but may actually increase the ranges for other fortificants.

## 5 Conclusions

Dietary inadequacies in key micronutrients remain important public health concerns in most LMICs. The results presented here in the contexts of Burkina Faso, Nigeria, and Senegal suggested that fortified bouillon cubes can be helpful in addressing these concerns, both in terms of reducing micronutrient inadequacies among young children and WRA, and also in reducing child mortality. However, while the marginal contributions of bouillon fortification were positive across most modeled fortification levels, the cost of bouillon fortification, as well as the degrees to which fortification reduced dietary micronutrient inadequacy or (where relevant) child mortality, varied by micronutrient, by level of fortification, and by country setting. Therefore, the cost‐effectiveness of bouillon fortification should *not* be considered a single number, but rather will be context‐specific and depend on the composition of premixes. In most settings and for most micronutrients examined here, at some level of fortification, the marginal benefits tended to decrease as fortification levels increased, some of which decreased to zero at some level of fortification. As a consequence, efficiency declined alongside reductions in marginal benefits; indeed, cost‐effectiveness became infinite when marginal benefits reached zero.

These results have important implications for policy. First, although efficiency is only one criterion for selecting micronutrient delivery vehicles and fortification levels (technical and commercial issues, affordability, equity, and sustainability also merit attention), it can help select the types and amounts of micronutrients to be included in premixes. For example, if “flat spots” in micronutrient‐specific nutritional and child mortality benefit curves exist, then policymakers generally should choose levels of fortification for each micronutrient that are below those at which the marginal benefits become quite small. Second, in all countries included in this analysis, the costs of achieving dietary adequacy were highest (by far) for iron and lowest for folic acid; vitamin A, vitamin B12, and zinc were in the intermediate zone in terms of cost‐effectiveness. In the absence of micronutrient‐specific social weighting schemes, these results can help prioritize the contents of premixes. Third, while efficiency can be an important guide in bouillon fortification program design, policymakers generally should not aim for the *most* cost‐effective levels of fortification for any given micronutrient; policy objectives for nutritional adequacy or child mortality reductions might not be met by these levels of fortificant concentrations. Therefore, avoiding *in*efficiencies might be a more useful decision‐making rule in choosing levels of fortificant concentrations than seeking absolute efficiency. Fourth, affordability might determine the upper bound on spending on fortification programs; once these limits are established, modeled cost‐effectiveness estimates can help select the most efficient programs among the subset of affordable programs. Finally, in bouillon and other LSFF fortification program policy discussions, it will be important to generate, package, and use the evidence on the benefits, costs, and cost‐effectiveness of fortification along a range of alternative fortification levels.

## Author Contributions

S.A.V., M.J., L.T., E.B., K.P.A., and R.E.‐S. designed the study and developed the methods. K.P.A., L.T., E.B., and M.J. analyzed the nutritional needs/benefits and program cost data, and provided the summary measures of nutritional benefits and program costs. S.A.V. and K.P.A. prepared a first draft of the paper and modified it based on reviewers’ comments. All authors contributed to the interpretation of simulation results, and all have read and approved the final manuscript.

## Funding

This work was supported, in part, by the Bill & Melinda Gates Foundation via a grant to Helen Keller International [INV‐007916].

## Conflicts of Interest

All authors report no conflicts of interest.

## Supporting information




**Supplementary Table**: nyas70137‐sup‐0001‐SuppMat.docx

## Footnotes

1 Moving forward in this paper, we will refer to bouillon generically, understanding that the vast majority of bouillon consumed in West Africa is in the form of cubes. (e.g., Anjorin, O. M., for Nigeria) [22].

2 Program “reach” is defined as having delivered a nonzero amount of a fortified or fortifiable food to a given household, regardless of household's nutritional status or the amount of a micronutrient delivered to it. We will use program reach in cost‐efficiency estimates, below.

3 Here and throughout, we use the term “diet” to refer to the estimated intake of foods at the household level.

4 For modeled fortificant absorption parameters, see Adams et al. [11, 12, 13].

5 See Table S1 for details regarding micronutrient‐specific effectiveness values.

6 One can, of course, simply add up the numbers of (say) WRA that achieve adequacy in each of the five micronutrients studied here, but doing so carries with it an implicit weight of “1” for each micronutrient, which may not capture the relative values to society.

7 Economic costs are comprised of out‐of‐pocket costs plus opportunity costs (e.g., value of un‐paid time dedicated to specific activities).

8 A 10‐year planning time horizon was adopted to ensure that all hypothetical programs, including those envisioning improvements to existing programs, have sufficient time to deliver micronutrients and generate the associated nutritional and mortality‐reducing benefits. No discount rate was applied.

9 See Adams et al. [11, 12, 13] for nutritional outcome results presented at the subnational level for each of the country case studies reported here.

10 Note that cost‐efficiency uses the number of individuals reached by a given program, regardless of their preprogram nutritional status, or whether or not the programs nutritional contributions caused a given individual to achieve nutritional adequacy. These estimates are reported in Table 3, in the context of bouillon fortified at 30% of Codex NRV in 2.5 g of product.

11 Similar but not identical patterns are reported elsewhere for young children [11, 12, 13]. Analyses were based on household‐level data, so similarities across beneficiary groups *within* given households are expected.

12 Pattern similarities were expected, since patterns of micronutrient inadequacies associated with alternative levels of bouillon fortification in WRA were similar to those for young children [11, 12, 13], and changes in prevalence of vitamin A and zinc inadequacies in children, and changes in the prevalence of inadequacies in folic acid in WRA, were used to predict changes in under‐5 child mortality.

13 Measuring fortification levels collectively in terms of % of Codex NRVs also complicates compatibility of results presented in Table 4.

14 There is a very large number candidate premix formulas if all possible combination micronutrients at different levels are considered. We present one combination here, and several alternative combinations below. Our choice of the 30% of NRV level of fortification were guided by labeling guidance provided by Codex, which, at that or higher levels, permits the declaration of “high in” on packaging and promotional materials (Codex Alimentarius Commission: Guidelines for Use of Nutrition Claims, CAC/GL 23‐1997, https://www.fao.org/4/y2770e/y2770e07.htm#fn22).

15 The results of sensitivity analyses associated with each of these tables are reported in Supplementary Material.

16 Program cost for each of the micronutrient‐specific program includes start‐up and operational costs for government and for industry, and, therefore, can be interpreted as stand‐alone fortification programs. We did not examine all possible combinations of the micronutrients in this analysis.

17 The cost of iron fortification depends greatly on the choice of fortificant. Micronized ferric pyrophosphate (FePP) was used in the results presented here. Costs would be substantially lower for (e.g.) electrolytic iron.

18 See Table S2 (Senegal), Table S3 (Nigeria), and Table S4 (Burkina Faso).

19 Recall that other existing LSFF programs also deliver these three micronutrients; the amounts delivered by them are accounted for in this analysis, hence these results should be interpreted at the marginal benefits of alternative bouillon fortification programs.

20 See Vosti et al. [18] for more evidence on bouillon program costs and potential cost burdens.

21 Patterns will be different for other forms of iron, for example, electrolytic iron.

22 For a review of the policy engagement approaches and achievements in each of the bouillon project countries, see Tarini et al. [49].
