# Supplementary material for: Cost‐Efficiency and Cost‐Effectiveness of Alternative Bouillon Fortification Programs: Evidence for Burkina Faso, Nigeria, and Senegal
Source: Ann N Y Acad Sci. 2025 Dec 9;1556(1):e70137. doi: 10.1111/nyas.70137 (PMC12917931; doi:10.1111/nyas.70137)
Supplement: Supplementary file 1 — Supplementary Table: nyas70137‐sup‐0001‐SuppMat.docx [file NYAS-1556-0-s001.docx]

**Cost-efficiency and Cost-effectiveness of Alternative Bouillon Cube Fortification Programs in Achieving Dietary Adequacy and Reducing Child Mortality:**

**Evidence from Burkina Faso, Nigeria, and Senegal**

**Online Supplementary Material**

This document contains information and research results to support the main paper.

**Table SM.1** reports the primary and secondary estimates (and their underlying assumptions and sources) used in the LiST modeling work regarding the effects on child mortality of achieving micronutrient adequacy in vitamin A, zinc, and folic acid. These assumptions were the same for each of the case study countries. The results reported in the main paper were based on the primary estimates; secondary estimates were used to generated some of the results reported in the sensitivity analyses (below).

**Table SM.1: Effectiveness Values Used in LiST Modeling**

| **Child-Lives Saved Estimates using LiST** | **Assumptions:**  **Vitamin A Effectiveness** | **Assumptions:**  **Zinc Effectiveness** | **Assumptions:**  **Folic Acid Effectiveness** |
| --- | --- | --- | --- |
| **Primary Estimates**  (results reported in main text) | **Definition:** Effect of achieving dietary adequacy on diarrhea mortality among children 6-59 months.  **Effectiveness estimate:**  0.74^1^ | **Definition:** Effect of achieving dietary adequacy on diarrhea and pneumonia mortality among children 12-59 months.  **Effectiveness estimates:**  Pneumonia: 0.064  Diarrhea: 0.068  Derived from meta-analysis of two studies,^2,3^ see Thompson et al. 2024^4^. Values account for risk of zinc deficiency among children based on prevalence of zinc inadequate intake defined by EFSA’s physiological requirement. | **Definition:** Effect of achieving dietary folate adequacy among pregnant women on fetal neural tube defects.  **Effectiveness estimate:**  0.41^5^ |
| **Secondary Estimates**  (results included in sensitivity analyses) | **Definition:** Effect of vitamin A supplementation on diarrhea mortality among children 6-59 months.  **Effectiveness estimate:**  0.53^6^ (LiST default) | **Definition:** Effect of achieving dietary adequacy on diarrhea and pneumonia mortality among children 12-59 months.  **Effectiveness estimate:**  Pneumonia: 0.14  Diarrhea: 0.15  Derived from meta-analysis of two studies^2,3^, see Thompson et al. 2024^4^. Values account for risk of zinc deficiency among children based on prevalence of zinc inadequate intake defined by EFSA’s physiological requirement. | **Definition:** Effect of achieving 400 mcg folic acid/day by a folic acid intervention among pregnant women on fetal neural tube defects.  **Effectiveness estimate:**  0.62^7^ |

**Table SM.2** (Senegal), **Table SM.3** (Nigeria), and **Table SM.4** (Burkina Faso) report the results of sensitivity analyses undertaken to assess the impacts on selected model results of uncertainty of key model parameters. Results based on our primary estimates are also included for comparison. More specifically, we assessed the effects of overestimates (20%+) and underestimates (20%$-) of fortification program costs (for programs delivering 30% of Codex NRV of each micronutrient separately, and for a program delivering 30% of Codex NRV of vitamin A, folic acid, and zinc, combined) on total program costs, costs per capita, cost per consumer reached, and cost per WRA reached.^[[1]](#footnote-1)^ We then estimated the range of fortified bouillon’s contributions (again assessed at 30% of Codex NRV) to effective coverage (% of WRA who achieved micronutrient adequacy), at 20%+ and 20%- the primary estimate. We then combine ranges of effective coverage and ranges of fortification program costs to estimate cost per WRA effectively covered; 20%-cost and 20%+ impact for an optimistic scenario, and 20%+cost and 20%-impact for a pessimistic scenario. Next, we assessed the effects of alternative LiST model assumptions on the estimated number of child-lives saved separately for vitamin A, folic acid, and zinc, and then for this trio of micronutrients jointly, using two levels of assumed impact (high and low) when generating secondary estimates. Finally, we combined the effects of uncertainty in program costs and child-lives saved to bookend the range of estimates of cost per child-life saved; low-cost plus primary estimates and high-cost plus secondary estimates.

**Table SM.2: Sensitivity Analyses: Cost, Effective Coverage, and Cost-efficiency, and Cost-effectiveness of Multi-Bouillon Fortification, 30% Codex in 2.5g, Senegal**

|  | **VA^1^** | **B12^1^** | **Folic Acid^1^** | **Iron^1^** | **Zinc^1^** |  | **Combined VA, zinc, folic acid** |
| --- | --- | --- | --- | --- | --- | --- | --- |
| **Total Program Costs** |  |  |  |  |  |  |  |
| Primary Estimate | $10,674,052 | $3,222,332 | $1,788,611 | $14,846,937 | $3,256,840 |  | $12,670,035 |
| 20%+ | $12,808,863 | $3,866,799 | $2,146,333 | $17,816,324 | $3,908,207 |  | $15,204,042 |
| 20%- | $8,539,242 | $2,577,866 | $1,430,889 | $11,877,549 | $2,605,472 |  | $10,136,028 |
|  |  |  |  |  |  |  |  |
| **Cost per Capita** |  |  |  |  |  |  |  |
| Primary Estimate | $0.07 | $0.020 | $0.011 | $0.09 | $0.021 |  | $0.08 |
| 20%+ | $0.08 | $0.024 | $0.014 | $0.11 | $0.025 |  | $0.10 |
| 20%- | $0.05 | $0.016 | $0.009 | $0.07 | $0.016 |  | $0.06 |
|  |  |  |  |  |  |  |  |
| **Cost per Individual Reached** |  |  |  |  |  |  |  |
| Primary Estimate | $0.08 | $0.023 | $0.013 | $0.10 | $0.023 |  | $0.09 |
| 20%+ | $0.09 | $0.027 | $0.015 | $0.13 | $0.028 |  | $0.11 |
| 20%- | $0.06 | $0.018 | $0.010 | $0.08 | $0.018 |  | $0.07 |
|  |  |  |  |  |  |  |  |
| **Cost per WRA Reached** |  |  |  |  |  |  |  |
| Primary Estimate | $0.30 | $0.09 | $0.05 | $0.42 | $0.09 |  | n/a^3^ |
| 20%+ | $0.36 | $0.11 | $0.06 | $0.50 | $0.11 |  | n/a |
| 20%- | $0.24 | $0.07 | $0.04 | $0.33 | $0.07 |  | n/a |
|  |  |  |  |  |  |  |  |
| **Bouillon’s Contribution to Effective Coverage^2^ (WRA)** |  |  |  |  |  |  |  |
| Primary Estimate | 19.84% | 7.11% | 18.66% | 4.38% | 22.33% |  | n/a |
| 20%+ | 23.81% | 8.53% | 22.40% | 5.25% | 26.80% |  | n/a |
| 20%- | 15.87% | 5.69% | 14.93% | 3.50% | 17.87% |  | n/a |
|  |  |  |  |  |  |  |  |
| **Cost per WRA Effectively Covered** |  |  |  |  |  |  |  |
| Primary Estimate | $1.35 | $1.14 | $0.24 | $8.52 | $0.37 |  | n/a |
| 20%-cost, 20%+impact | $0.90 | $0.76 | $0.16 | $5.68 | $0.24 |  | n/a |
| 20%+cost, 20%-impact | $2.03 | $1.71 | $0.36 | $12.79 | $0.55 |  | n/a |
|  |  |  |  |  |  |  |  |
| **Child-Lives Saved** |  |  |  |  |  |  |  |
| Primary Estimate^4^ | 2,137 | n/a | 1,314 | n/a | 498 |  | 3,949 |
| Secondary Estimate^4^ | 1,553 | n/a | 48^5^ | n/a | 787 |  | 2,379 |
|  |  |  |  |  |  |  |  |
| **Cost per Child-life Saved** |  |  |  |  |  |  |  |
| Primary Estimate | $4,995 | n/a | $1,361 | n/a | $6,540 |  | $3,208 |
| 20%- costs, high impact^6^ estimates |  |  |  |  |  |  | $2,391 |
| 20%+ costs, low impact^6^ estimates |  |  |  |  |  |  | $7,278 |

^1^ VA, Retinyl Palmitate- 250,000 IU/g (dry); B12, Vit. B-12 0.1% WS; Folic acid, folic acid; Iron, micronized ferric pyrophosphate; Zinc, Zinc oxide. For fortificant absorption parameters, see Adams et al. (2024).^9^ All values are reported in 2021 USD.

^2^ Changes in apparent dietary micronutrient adequacy attributable to fortified bouillon, considering baseline diets and existing fortification programs.

^3^ n/a, not applicable.

^4^ The primary and secondary estimates are determined by the effectiveness values in the LiST model for any vehicle that delivers vitamin A, zinc, and folic acid. ‘Effectiveness’ in LiST is the estimated risk reduction in child mortality associated with achieving dietary adequacy.

^5^The secondary estimate for lives saved due to folic acid assumes effective coverage is the proportion of women achieving 400mcg folic acid per day from fortification, which is recommended during pregnancy for prevention of neural tube defects. This value does not consider intake of folate from other food sources, which would contribute to dietary folate intake. Therefore, this estimate is much lower than that of the primary estimate, which assumes effective coverage equates the proportion of women reaching or exceeding the EAR for folate through fortification in the presence of dietary intake of folate from other foods.

^6^High impact refers to the combination of estimates that yield the greatest number of child-lives saved (4,239), and low impact refers to the combination of estimates that yield the smallest number of child-lives saved (2,089), based on the range of effectiveness values within LiST for vitamin A, zinc and folic acid.

**Table SM.3: Sensitivity Analyses: Cost, Effective Coverage, and Cost-efficiency, and Cost-effectiveness of Multi-Bouillon Fortification, 30% Codex in 2.5g, Nigeria**

|  | **VA^1^** | **B12^1^** | **Folic Acid^1^** | **Iron^1^** | **Zinc^1^** |  | **Combined VA, zinc, folic acid** |
| --- | --- | --- | --- | --- | --- | --- | --- |
| **Total Program Costs** |  |  |  |  |  |  |  |
| Primary Estimate | $227,660,707 | $45,772,627 | $10,780,186 | $329,303,892 | $46,570,343 |  | $276,333,911 |
| 20%+ | $273,192,848 | $54,927,153 | $12,936,224 | $395,164,670 | $55,884,412 |  | $331,600,694 |
| 20%- | $182,128,565 | $36,618,102 | $8,624,149 | $263,443,113 | $37,256,275 |  | $221,067,129 |
|  |  |  |  |  |  |  |  |
| **Cost per Capita** |  |  |  |  |  |  |  |
| Primary Estimate | $0.117 | $0.024 | $0.006 | $0.170 | $0.024 |  | $0.143 |
| 20%+ | $0.141 | $0.028 | $0.007 | $0.204 | $0.029 |  | $0.171 |
| 20%- | $0.094 | $0.019 | $0.004 | $0.136 | $0.019 |  | $0.114 |
|  |  |  |  |  |  |  |  |
| **Cost per Consumer Reached** |  |  |  |  |  |  |  |
| Primary Estimate | $0.119 | $0.024 | $0.006 | $0.173 | $0.024 |  | $0.145 |
| 20%+ | $0.143 | $0.029 | $0.007 | $0.207 | $0.029 |  | $0.174 |
| 20%- | $0.096 | $0.019 | $0.005 | $0.138 | $0.020 |  | $0.116 |
|  |  |  |  |  |  |  |  |
| **Cost per WRA Reached** |  |  |  |  |  |  |  |
| Primary Estimate | $0.51 | $0.10 | $0.024 | $0.73 | $0.10 |  | n/a^3^ |
| 20%+ | $0.61 | $0.12 | $0.029 | $0.88 | $0.12 |  | n/a |
| 20%- | $0.41 | $0.08 | $0.019 | $0.59 | $0.08 |  | n/a |
|  |  |  |  |  |  |  |  |
| **Bouillon’s Contribution to Effective Coverage^2^ (WRA)** |  |  |  |  |  |  |  |
| Primary Estimate | 15.47% | 19.52% | 13.16% | 4.18% | 12.71% |  | n/a |
| 20%+ | 18.56% | 23.43% | 15.79% | 5.01% | 15.25% |  | n/a |
| 20%- | 12.37% | 15.62% | 10.53% | 3.34% | 10.17% |  | n/a |
|  |  |  |  |  |  |  |  |
| **Cost per WRA Effectively Covered** |  |  |  |  |  |  |  |
| Primary Estimate | $3.22 | $0.51 | $0.18 | $17.25 | $0.80 |  | n/a |
| 20%-cost, 20%+impact | $2.15 | $0.34 | $0.12 | $11.50 | $0.53 |  | n/a |
| 20%+cost, 20%-impact | $4.83 | $0.77 | $0.27 | $25.87 | $1.20 |  | n/a |
|  |  |  |  |  |  |  |  |
| **Child-Lives Saved** |  |  |  |  |  |  |  |
| Primary Estimate^4^ | 136,022 | n/a | 38,271 | n/a | 40,997 |  | 215,290 |
| Secondary Estimate^4^ | 99,168 | n/a | 964^5^ | n/a | 54,389 |  | 154,328 |
|  |  |  |  |  |  |  |  |
| **Cost per Child-life Saved** |  |  |  |  |  |  |  |
| Primary Estimate | $1,674 | n/a | $282 | n/a | $1,136 |  | $1,284 |
| 20%- costs, high impact^6^ estimates |  |  |  |  |  |  | $967 |
| 20%+ costs, low impact^6^ estimates |  |  |  |  |  |  | $2,353 |

^1^ VA, Retinyl Palmitate- 250,000 IU/g (dry); B12, Vit. B-12 0.1% WS; Folic acid, folic acid; Iron, micronized ferric pyrophosphate; Zinc, Zinc oxide. For fortificant absorption parameters, see Adams et al. (2024).^10^ All values are reported in 2021 USD.

^2^ Changes in apparent dietary micronutrient adequacy attributable to fortified bouillon, considering baseline diets and existing fortification programs.

^3^ n/a, not applicable.

^4^ The primary and secondary estimates are determined by the effectiveness values in the LiST model for any vehicle that delivers vitamin A, zinc, and folic acid. ‘Effectiveness’ in LiST is the estimated risk reduction in child mortality associated with achieving dietary adequacy.

^5^The secondary estimate for lives saved due to folic acid assumes effective coverage is the proportion of women achieving 400mcg folic acid per day from fortification, which is recommended during pregnancy for prevention of neural tube defects. This value does not consider intake of folate from other food sources, which would contribute to dietary folate intake. Therefore, this estimate is much lower than that of the primary estimate, which assumes effective coverage equates the proportion of women reaching or exceeding the EAR for folate through fortification in the presence of dietary intake of folate from other foods.

^6^High impact refers to the combination of estimates that yield the greatest number of child-lives saved (228, 681), and low impact refers to the combination of estimates that yield the smallest number of child-lives saved (140,937), based on the range of effectiveness values within LiST for vitamin A, zinc and folic acid.

**Table SM.4: Sensitivity Analyses: Cost, Effective Coverage, and Cost-efficiency, and Cost-effectiveness of Multi-Bouillon Fortification, 30% Codex in 2.5g, Burkina Faso**

|  | **VA^1^** | **B12^1^** | **Folic Acid^1^** | **Iron^1^** | **Zinc^1^** |  | **Combined VA, zinc, folic acid** |
| --- | --- | --- | --- | --- | --- | --- | --- |
| **Total Program Costs** |  |  |  |  |  |  |  |
| Primary Estimate | $8,178,862 | $2,664,504 | $1,603,534 | $11,266,845 | $2,690,040 |  | $9,655,913 |
| 20%+ | $9,814,635 | $3,197,405 | $1,924,241 | $13,520,214 | $3,228,048 |  | $11,587,095 |
| 20%- | $6,543,090 | $2,131,603 | $1,282,827 | $9,013,476 | $2,152,032 |  | $7,724,730 |
|  |  |  |  |  |  |  |  |
| **Cost per Capita** |  |  |  |  |  |  |  |
| Primary Estimate | $0.04 | $0.013 | $0.008 | $0.06 | $0.013 |  | $0.05 |
| 20%+ | $0.05 | $0.016 | $0.010 | $0.07 | $0.016 |  | $0.06 |
| 20%- | $0.03 | $0.011 | $0.006 | $0.05 | $0.011 |  | $0.04 |
|  |  |  |  |  |  |  |  |
| **Cost per Individual Reached** |  |  |  |  |  |  |  |
| Primary Estimate | $0.05 | $0.016 | $0.010 | $0.07 | $0.016 |  | $0.06 |
| 20%+ | $0.06 | $0.019 | $0.012 | $0.08 | $0.020 |  | $0.07 |
| 20%- | $0.04 | $0.013 | $0.008 | $0.05 | $0.013 |  | $0.05 |
|  |  |  |  |  |  |  |  |
| **Cost per WRA Reached** |  |  |  |  |  |  |  |
| Primary Estimate | $0.21 | $0.07 | $0.04 | $0.28 | $0.07 |  | n/a^3^ |
| 20%+ | $0.25 | $0.08 | $0.05 | $0.34 | $0.08 |  | n/a |
| 20%- | $0.17 | $0.05 | $0.03 | $0.23 | $0.05 |  | n/a |
|  |  |  |  |  |  |  |  |
| **Bouillon’s Contribution to Effective Coverage^2^ (WRA)** |  |  |  |  |  |  |  |
| Primary Estimate | 7.62% | 5.70% | 15.48% | 1.60% | 6.38% |  | n/a |
| 20%+ | 9.14% | 6.85% | 18.57% | 1.92% | 7.65% |  | n/a |
| 20%- | 6.09% | 4.56% | 12.38% | 1.28% | 5.10% |  | n/a |
|  |  |  |  |  |  |  |  |
| **Cost per WRA Effectively Covered** |  |  |  |  |  |  |  |
| Primary Estimate | $2.23 | $0.97 | $0.22 | $14.59 | $0.88 |  | n/a |
| 20%-cost, 20%+impact | $1.49 | $0.65 | $0.14 | $9.73 | $0.58 |  | n/a |
| 20%+cost, 20%-impact | $3.35 | $1.46 | $0.32 | $21.89 | $1.31 |  | n/a |
|  |  |  |  |  |  |  |  |
| **Child-Lives Saved** |  |  |  |  |  |  |  |
| Primary Estimate^4^ | 4,536 | n/a | 1,973 | n/a | 1,112 |  | 7,621 |
| Secondary Estimate^4^ | 3,306 | n/a | 0^5^ | n/a | 1,508 |  | 4,815 |
|  |  |  |  |  |  |  |  |
| **Cost per Child-life Saved** |  |  |  |  |  |  |  |
| Primary Estimate | $1,803 | n/a | $813 | n/a | $2,419 |  | $1,267 |
| 20%- costs, high impact estimates^6^ |  |  |  |  |  |  | $964 |
| 20%+ costs, low impact estimates^6^ |  |  |  |  |  |  | $2,623 |

^1^ VA, Retinyl Palmitate- 250,000 IU/g (dry); B12, Vit. B-12 0.1% WS; Folic acid, folic acid; Iron, micronized ferric pyrophosphate; Zinc, Zinc oxide. For fortificant absorption parameters, see Adams et al. (2024).^11^ All values are reported in 2021 USD.

^2^ Changes in apparent dietary micronutrient adequacy attributable to fortified bouillon, considering baseline diets and existing fortification programs.

^3^ n/a, not applicable.

^4^ The primary and secondary estimates are determined by the effectiveness values in the LiST model for any vehicle that delivers vitamin A, zinc, and folic acid. ‘Effectiveness’ in LiST is the estimated risk reduction in child mortality associated with achieving dietary adequacy.

^5^The secondary estimate for lives saved due to folic acid assumes effective coverage is the proportion of women achieving 400mcg folic acid per day from fortification, which is recommended during pregnancy for prevention of neural tube defects. This value does not consider intake of folate from other food sources, which would contribute to dietary folate intake. Therefore, this estimate is much lower than that of the primary estimate, which assumes effective coverage equates the proportion of women reaching or exceeding the EAR for folate through fortification in the presence of dietary intake of folate from other foods.

^6^High impact refers to the combination of estimates that yield the greatest number of child-lives saved (8,017), and low impact refers to the combination of estimates that yield the smallest number of child-lives saved (4,418), based on the range of effectiveness values within LiST for vitamin A, zinc and folic acid.

**References**

^1^ Engle-Stone R, Perkins A, Clermont A, et al. Estimating Lives Saved by Achieving Dietary Micronutrient Adequacy, with a Focus on Vitamin A Intervention Programs in Cameroon. *The Journal of Nutrition*. 2017;147(11):2194S-2203S.

^2^ Tsang BL, Holsted E, McDonald CM, et al. Effects of foods fortified with zinc, alone or cofortified with multiple micronutrients, on health and functional outcomes: a systematic review and meta-analysis. *Advances in Nutrition*. 2021;12(5):1821-1837.

^3^ Mayo‐Wilson E, Junior JA, Imdad A, et al. Zinc supplementation for preventing mortality, morbidity, and growth failure in children aged 6 months to 12 years of age. *Cochrane Database of Systematic Reviews*. 2014(5).

^4^ Thompson, L., Becher, E., Adams, K. P., Haile, D., Walker, N., Tong, H., Vosti, S. A., & Engle-Stone, R. (2024, Jul). Modeled impacts of bouillon fortification with micronutrients on child mortality in Senegal, Burkina Faso, and Nigeria. *Ann N Y Acad Sci*, 1537(1), 82-97. <https://doi.org/10.1111/nyas.15174>

^5^ Blencowe H, Kancherla V, Moorthie S, Darlison MW, Modell B. Estimates of global and regional prevalence of neural tube defects for 2015: a systematic analysis. *Annals of the New York Academy of Sciences*. 2018;1414(1):31-46.

^6^ Imdad A, Yakoob MY, Sudfeld C, Haider BA, Black RE, Bhutta ZA. Impact of vitamin A supplementation on infant and childhood mortality. *BMC Public Health*. 2011;11 Suppl 3(Suppl 3):S20.

^7^ Luo H, Brown KH, Stewart CP, et al. Review of Existing Models to Predict Reductions in Neural Tube Defects Due to Folic Acid Fortification and Model Results Using Data from Cameroon. *Advances in Nutrition*. 2021;12(6):2401-2414

^8^ Vosti, S. A., Jarvis, M., Anjorin, O. M., Engle-Stone, R., Beye, M., Ishaya, F., Koudougou, K., Oni, B., Somda, H., & Adams, K. P. (2024). The costs and the potential allocation of costs of bouillon fortification: The cases of Nigeria, Senegal, and Burkina Faso. *Ann NY Acad Sci*., 1541, 181–201. <https://doi.org/10.1111/nyas.15234>

^9^Adams, K. P., Vosti, S. A., Tarini, A., Beye, M., Pachón, H., Kiselova, S., and Engle-Stone, R. (2024). The potential contributions of bouillon fortification to meeting micronutrient requirements among women and preschool children in Senegal: A modeling study using household consumption and expenditure survey data. 2024. *Annals of the New York Academy of Sciences*. 1537, 98-112. http//doi.org/10.1111/nyas.15156

^10^ Adams, K. P., Vosti, S. A., Becher, E., Ishaya, F., and Engle-Stone, R. (2024). Bouillon fortification as a strategy to address inequities in micronutrient adequacy of diets in Nigeria. *Ann NY Acad Sci.*, First published: 10 September 2024, <https://doi.org/10.1111/nyas.15207>

^11^ Adams, K. P., Vosti, S. A., Somé, J. S. Tarini, A., Bercher, E., Koudougou, K., and Engle-Stone, R. (2024). Micronutrient-fortified bouillon as a strategy to improve the micronutrient adequacy of diets in Burkina Faso. *Annals of the New York Academy of Sciences*. 1536, 135–150. <https://doi.org/10.1111/nyas.15155>.

1. For details regarding bouillon fortification cost calculations, see Vosti et al. 2024.^8^ [↑](#footnote-ref-1)
